# Targeting Wnt Signaling in the Tumor Immune Microenvironment to Enhancing EpCAM CAR T-Cell therapy

**DOI:** 10.3389/fphar.2021.724306

**Published:** 2021-11-01

**Authors:** Weizhen Li, Yang Zhou, Zhongen Wu, Yaoping Shi, Enming Tian, Yingqi Zhu, Tao Wang, Wei Dou, Xiangjing Meng, Ming Chen, Bo Zhai, Di Zhu

**Affiliations:** ^1^ Department of Laboratory Medicine, Sixth Affiliated Hospital of Yangzhou University, Taizhou, China; ^2^ Department of Laboratory Medicine, Affiliated Taixing Hospital of Bengbu Medical College, Taizhou, China; ^3^ School of Pharmacy, Fudan University, Shanghai, China; ^4^ Department of Interventional Oncology, Renji Hospital, Shanghai Jiao Tong University School of Medicine, Shanghai, China; ^5^ Shanghai University of Traditional Chinese Medicine, Shanghai, China; ^6^ Shandong Academy of Pharmaceutical Science, Jinan, China; ^7^ State Key Laboratory of Oncogenes and Related Genes, Shanghai Cancer Institute, Renji Hospital, Shanghai Jiao Tong University School of Medicine, Shanghai, China; ^8^ Shanghai Engineering Research Center of ImmunoTherapeutics, Fudan University, Shanghai, China; ^9^ Department of Pharmacology, School of Basic Medical Sciences, Fudan University, Shanghai, China

**Keywords:** chimeric antigen receptor T cells, Wnt signaling, BCL9, tumor immune microenvironment, EpCAM

## Abstract

Colorectal cancer (CRC) patients are still lacking viable treatments. Chimeric antigen receptor (CAR) T cells have shown promise in hematologic malignancies, but their efficacy in solid tumors has been limited due to the immunosuppressive tumor microenvironment. We found that cancer antigen- EpCAM expression increased in the metastatic stage compared with the primary stage in cancers and the activation of Wnt and TGFβ pathways was positively correlated with EpCAM expression in multiple cancers, including colorectal cancer. We constructed CAR T cells targeting EpCAM that successfully showed selective cytotoxicity in highly EpCAM-expressing cancer cell lines. The combination of EpCAM CAR-T with the Wnt inhibitor-hsBCL9_CT_-24 displayed synergetic effect against EpCAM-positive colon cells *in vitro* and also *in vivo*. A mechanistic study showed that hsBCL9_CT_-24 treatment could modulate the tumor environment and improve infiltration of T cells, while possibly promoting the effector T cells at the early stages and postponing the exhaustion of CAR T cells at advanced stages. Overall, these results demonstrated that the combination of EpCAM CAR T-cell therapy with the Wnt inhibitor can overcome the limitations of CAR T cells in treating solid tumors.

## Introduction

Colorectal cancer (CRC) is the world’s third most common malignancy, ranking fourth in the world among cancer deaths. The number of people with colorectal cancer is expected to increase by 60% by 2030, with the number of new cases expected to exceed two million per year and the number of deaths to exceed 1.1 million (Bray et al., 2018). In recent years, with the emergence of new chemotherapy and small molecule drugs in clinical applications, metastasis and advanced colorectal cancer can be treated, but there is still a lack of effective treatment for colorectal cancer, and the strong side effects of the drugs make it difficult to improve the quality of life for patients (McQuade et al., 2017).

Adoptive cell therapy (ACT) is an efficient and personalized cancer treatment that uses T cells that target tumor antigens to perform natural functions to kill tumor cells. The therapy involves *in vitro* amplification, modification, activation, and re-transmission of autoimmune cells to patients; the immune cells can enhance the lethal suppression of tumor cells (Goff et al., 2016). Compared with immunotherapy that blocks checkpoints, secondary cell therapy has fewer toxic side effects, is more tolerant, and does not produce drug resistance. Here, we explored chimeric antigen receptor (CAR) T-cell therapy by building suitable CAR T cells to examine their effect on colon cancer tumor cells and the relevant influencing factors.

Chimeric antigen receptors (CARs) are engineered receptors that transfer a specific antibody to immune-effect cells (T cells). CARs are designed to redirect the patient or donor’s T cells to specifically target and kill tumor cells. There are four generations of CAR T cells; in general, a CAR consists of three parts: an extracellular antigen recognition domain from the single-chain fragment variant (scFv) of the antibody, the intracellular T-cell activation domain and the transmembrane domain between them (Ramos and Dotti, 2011). The first generation of CARs were single-stranded antibodies (CD3- or Fc- RI) that connected to an immune receptor tyrosine to activate the base sequence (ITAM) (Gross et al., 1989; Eshhar et al., 1993). In the second generation of CARs, the signal transduction region was joined by a co-iris (CM1), such as CD28 (Finney et al., 1998). The third generation adds another co-iris (CM2), such as CD134 or CD137 (Zhong et al., 2010). The fourth generation adds key cytokines such as IL-12 on the basis of the second and third generations (Chmielewski and Abken, 2015).

EpCAM is a tumor-related antigen first found in colon cancer tissue that is one of the main surface antigens of human colon cancers (Herlyn et al., 1979). It is in essence a membrane protein with adhesion function. Research has shown that it is highly expressed in tumor tissues and is also expressed in normal cells (Trzpis et al., 2007; Patriarca et al., 2012). Studies have also shown that the EpCAM extracellular domain can promote colon cancer cell migration, proliferation, and tumor growth by activating EGFR and downstream ERK1/2 signaling (Liang et al., 2018). EpCAM is a target of the Wnt/β-catenin pathway, and this signaling pathway controls the proliferation of hepatic stem cells (Ishiguro et al., 2020). Prognostic analyses have shown an inverse correlation between EpCAM, Wnt/β-catenin expression and patient prognosis. The activation of tumor-intrinsic Wnt/β-catenin pathway frequently contributes to poor infiltration of T cell across most human cancers (Luke et al., 2019).

The complex immune microenvironment of solid tumors is the main factor affecting the immune effect. The immunosuppressive cells and factors are adverse to recruitment of effector immune cells and are implicated in their dysfunction, as they may affect the uniform immersion and persistence of CAR-T cells in the tumor (Rahir and Moser, 2012). These challenges must be overcome by various methods (e.g., immunomodulation microenvironment of the solid tumor) (Roessler et al., 2014; Beavis et al., 2016). In addition, the heterogeneity of tumor cells and the lack of cancer-specific antigens is another factor affecting the immune response in solid tumors (Roessler et al., 2014). Finding the proper target or target combination will determine the final treatment outcome.

Heterogeneous and complex immune microenvironments of tumors are the key factors limiting CAR-T efficacy of cancer treatment. The combination of specific CAR T-cell therapy and immunomodulators provides an opportunity to solve the difficulties of solid tumor treatment. In melanomas, the Wnt signaling pathway is activated, preventing T cells from infiltrating tumor cells, thereby reducing the effectiveness of immunotherapy (Spranger et al., 2015). By inhibiting the Wnt signaling pathway of the tumor, T-cell infiltration can be promoted to improve the immune response (Ganesh et al., 2018). M Feng et al. identified a peptide, hsBCL9_CT_-24, which inhibits β-catenin/BCL9 interaction and exhibits potent anti-tumor effects, including promotion of cytotoxic T cells infiltration in tumors accompanied by an increase in dendritic cells (DCs) and a reduction in regulatory T cells (Treg) (Feng et al., 2019). Here, we constructed a second-generation CAR targeting EpCAM and investigated whether the EpCAM CAR T synergized by hsBCL9_CT_-24 could enhance the antitumor activity against human CRC. Furthermore, we examined whether application of hsBCL9_CT_-24 could facilitate the infiltration of CAR-T and ameliorate its function.

## Materials and Methods

### EpCAM Expression Analysis

From the Human Protein Atlas (http://www.proteinatlas.org/), *EPCAM* RNA-seq data in normal tissues generated by the Genotype-Tissue Expression (GTEx) project is downloaded. The bar plot is generated in GraphPad Prism 8.

*EPCAM* expression in cancers and tumor vs matched normal tissue is ploted in GEPIA (http://gepia.cancer-pku.cn/) which includes the RNA sequencing expression data of 8,587 normal and 9,736 tumor samples from the GTEx projects and the TCGA.

IHC pictures of normal tissues and tumor staining EpCAM in patients with colorectal cancer (patient id: 1958) and stomach cancer (patient id: 2105) are obtained from the Human Protein Atlas (https://www.proteinatlas.org/).

### GEO and TCGA Datasets

Gene Expression Omnibus (GEO) datasets GSE81558 were based on the Affymetrix Human Gene Expression Array expression BeadChip platform and comprised of nine non-tumoral colorectal samples, 23 primary colorectal tumor samples, and 19 CRC-LM samples. Its raw data was downloaded from the GEO database (www.ncbi.nlm.nih.gov/geo/) and analyzed. The Cancer Genome Atlas (TCGA) datasets, including COAD and READ, were downloaded from cBioPortal (http://www.cbioportal.org/). And according to gene median expression level, CRC samples were divided into high and low expression groups.

### Cell Culture

Human CRC cell lines (HCT116, SW480 and RKO) as well as human lung adenocarcinoma cell line A549 and normal human pancreatic epithelial cell line HPDE6-C7 were purchased from ATCC (Manassas, VA) and cultured in Dulbecco’s modified Eagle’s medium (DMEM, Hyclone, Gelifescience, America) with 10% FBS (Gibco, Life Technologies, America) and 1% penicillin/streptomycin (Gibco, Life Technologies, America). T cells were isolated from fresh vein blood of healthy donors and incubated in complete RPMI medium supplemented with 200 IU/ml recombinant human rhIL-2 (PeproTech, America). In co-culture experiments, rhIL-2 was free. All cells were cultured in a cell culture incubator with humidified atmosphere and 5% CO_2_ at 37°C.

### Construction of Anti-EpCAM-CAR Lentiviral Vector

The construct of anti-EpCAM-CAR sequentially includes an anti-human EpCAM single-chain variable fragment sequence [scFv (Genebank AJ564232.1)], CD8 alpha hinge and transmembrane domains, CD28 co-stimulatory domain and the CD3ζ intracellular domain. This EpCAM-specific CAR was inserted into a PMC245-97812 lentiviral vector (ProMab Biotachnologies, Inc.) and the whole sequence was confirmed by direct sequencing. As control vector, green fluorescent protein (GFP) coding sequence took the place of anti-EpCAM scFv to generate membrane-bound GFP (mGFP) CAR. To produce lentiviral particles, PMC245-97812-based plasmid was transduced into 293T cells with the packaging plasmids psPAX2 (Ge Healthcare) and PMD2.0G (Hanbio, China). After 48 and 72 h, the supernatants containing the lentivirus particles were collected, filtered, and concentrated at 82700 g for 120 min. Finally, the concentrated lentivirus of about 10^8–9^ IFU/mL were stored at −80°C.

### Generation of CAR-T Cells

For generating CAR-T cells by lentivirus-transduction, firstly, from fresh vein blood of healthy donors, human peripheral blood mononuclear cells (PBMCs) were collected as buffy coats through density gradient centrifugation using Ficoll-Paque (Ge Healthcare, Sweden). Then after filtration, resting CD3+ T cells were enriched from PBMCs by depleting non CD3+ T cells using MojoSort™ Human CD3 T Cell Isolation Kit (Biolegend, 480022, United States). The isolated cells were cultured in 6-well plates with above complete RPMI medium, with addition of anti-human CD3/CD28 immune magnetic beads (Miltenyi Biotech, T cell activation/expansion kit, human, 130-091-441, Germany) to stimulate at a cell-to-bead ratio of 2:1. After 4 days of stimulation, mGFP CAR- or anti- EpCAM CAR-lentivirus supernatant were used to transduce those cells at a multiplicity of infection (MOI) of 5, with polybrene at a final concentration of 5 μg/ml in serum free RPMI medium containing 200 IU/ml rhIL-2. Then the plates were centrifuged at 37°C and 200 g for 60 min before culture. After 6 h culture, the medium was replaced by fresh complete RPMI medium. Two days later, beads were removed, and after the validation of anti- EpCAM CAR expression, the lentivirus-infected T cells were continuously expanded or used for experiments *in vitro* or *in vivo*. Human PBMC was donated by Weizhen Li and Zhongen Wu who provided their written informed consent. All the CAR-T cells were used *in vitro* and in animal only. Studies didn’t involve Human Participants. Human PBMC collected was approved by ethic committee of Minhang Affiliated hospital (2019-Pijian-010-01 K).

### Flow Cytometric Analysis

To detect CAR expression on cell surface, recombinant biotinylated protein L (Thermo Fisher, United States ) was used to bind to scFv for 30 min at 4°C, followed by phycoerythrin (PE) -conjugated streptavidin (eBioscience, Lot4306318, United States). To detect EpCAM expression on target cells, APC- conjugated anti-human CD326 (EpCAM) antibody (Biolegend, Cat369809, United States) was used. To detect phenotype of T cells, APC-conjugated anti-human CD3 antibody (Invitrogen, Lot1987999, United States), FITC-conjugated anti-human CD4 antibody (eBioscience, Lot 4309494, United States), PE-Cy7-conjugated anti-human CD8 antibody (invitrogen, Lot2071279, United States) were used. To analyse memory phenotype of the CAR-T cells, PerCP-conjugated anti-CCR7 antibody (Biolegend, Cat353241, United States) and PE-conjugated anti-CD45RA antibody (Biolegend, Cat304108, United States) were used. Cells were stained with antibodies in dark for 30 min at 4°C and washed with fluorescence activated cell sorting (FACS) buffer (PBS, 2% BSA) before and after staining. Flow cytometry data were acquired with a CytoFlex S (Beckman Coulter, United States) and analysed by Flowjo V10.

### Western Blot Analysis

For detecting the EpCAM expression on target cell lines, whole-cell protein was extracted by lysing cells on ice for 30 min using RIPA (Beyotime, China) with 0.1 mM PMSF (Sigma, United States). Cell lysates were centrifuged at 12000 rpm for 15 min at 4°C, the supernatant was collected and determined the concentration by a BCA kit (Beyotime, China). Following loading equal amounts of samples (25 μg) on 10% SDS-PAGE, proteins were separated then transferred onto a PVDF membranes (Immobilon-P, United States). After blocking, each membrane was incubated with one primary antibody against target overnight at 4°C. The rabbit anti-human EpCAM antibody (1:1000, Proteintech, Lot00044849, United States) was used to detect EpCAM expression on a set of cells and rabbit anti-human *β*-Actin antibody (1:1000, Proteintech, Cat 20536-1-AP, United States) as the internal control. After wash, membranes were then incubated with a goat anti-rabbit secondary antibody (1:2000, Proteintech, United States) for 40 min at room temperature. For detecting T-bet and FoxO1 in the cytoplasm and nucleus, nuclear and cytoplasmic proteins were extracted according to the manufacturer instructions that came with the nuclear and cytoplasmic protein extraction kit (Beyotime, China). Rabbit anti-human T-bet mAb (1:1000, Proteintech, Lot 00019612, United States) and Rabbit anti-human FoxO1 mAb (1:1000, Proteintech, Lot00056119, United States) were used to detect T-bet and FoxO1 in the nucleus and cytoplasm respectively. Rabbit anti-human Histone H3 antibody (1:1000, Cell Signaling Technology, #4499T, United States) was chosen to detect H3 in the nucleus as a nucleus internal control. Finally, the ECLsystem (Beyotime, China) was used to detect the target proteins and Tanon5200 (China) for visualization.

### Quantitative Reverse Transcription PCR

The total RNA of T cells collected from coculture with HCT116 cells for 24 h, 48 h or longer time *in vitro* or tumor tissue obtained from animal experiment was extracted using Trizol and the following qRT-PCR was carried out according to the protocol of Takara (RR036Q, RR820A) by CFX96TM Real-Time System (Bio-Rad). In this study, the following primer pairs were used:

*MMP7*: F-5′TGTATGGGGAACTGCTGACA3′, R-5′GCGTTCATCCTCATCGAAGT3’;
*TGF-β*: F-5′CAATTCCTGGCGATACCTCAG3′, R-5′GCACAACTCCGGTGACATCAA3’;
*CXCL10*: F-5′AGTGGCATTCAAGGAGTACCT3′, R-5′TGATGGCCTTCGATTCTGGA3’;
*IFN-γ*: F-5′TCGGTAACTGACTTGAATGTCCA3′, R-5′TCGCTTCCCTGTTTTAGCTGC3′
*BLIMP1*: F-5′GCAGAACGGCAAGATCAAGT3′, R-5′AAGCCCTTGTTGCAAGTCTG3’;
*ID2*: F-5′CTGGACTCGCATCCCACTAT3′, R-5′TATCCGTGTTGAGGGTGGTC3’;
*TCF1*: F-5′CTGCACATGCAGCTATACCC3′, R-5′GCACTGTCATCGGAAGGAAC3’;
*NOTCH1*: F-5′GTACAAGTGCGACTGTGACC3′, R-5′GCACACTCGTTGATGTTGGT3’;
*EOMES*: F-5′GCCTCTGTGGCTCAAATTCC3′, R-5′CACATTGTAGTGGGCAGTGG3’.

### Real-Time Cell Assay

To determine the cytotoxic activity of CAR-T cells, RTCA was performed. First, a 2 × 8-well E-Plate (ACEA Biosciences, United States) was added 50 μl culture media (RPMI medium supplemented with 10% FBS and 1% penicillin/streptomycin) per well, then the background impedance was scanned and data was recorded as the basal cell index (CI). Then, 5000 target cells in 50 μl culture medium were seeded in each well and after 30 min of deposition, the E-Plate was transferred to the xCELLigence system. Data recording was acquired every 15 min. When the target cells reached a logarithmic growth phase, effector cells in a volume of 50 μl were added into each well at different effector/target (E: T) ratios, in duplicate. Then the E-Plate was transferred back and data recording was restarted. The cytotoxic activity of CAR-T cells was monitored grounded on the viability of the attached target cells and reflected by CI value. Here effector cells produced low baseline level for their lack of tight surface adhesion over the gold electrodes, but their addition and following cytolytic activity caused rounding up and detaching of the adherent target cells, consequently reduced CI value. The system was also applied to test the effect of hsBCL9_CT_-24 peptide on the viability of tumor cells by adding different concentration and to detect the synergetic effect of hsBCL9_CT_-24 with EpCAM CAR T.

### Cytokine Release Analysis by ELISA

Effector cells were co-cultured with target cells (5 × 10^3^ cells each base) in RPMI medium supplemented with 10% FBS and 1% penicillin/streptomycin at E:T ratio of 4:1 or 8:1 in 96-well plates in triplicate. In the combination treatment, hsBCL9_CT_-24 was added simultaneously. After 48 h of co-culture and through centrifugation at 300 g for 5 min, the cell-free supernatant was gained and the level of cytokine IFN-γ was quantified by a human ELISA kit according to the manufacturer’s instruction (Multiscience, China).

### Transwell Migration Assay

To investigate the effect of hsBCL9_CT_-24 on migration of CAR-T cells, *in vitro* cell migration assay was performed. Target tumor cells were seeded in 24-well plates (2 × 10^5^ cells/well in 500 µl complete RPMI medium) and cultured for 10–18 h until they are adhered to the bottom. Then 2 × 10^5^ CAR-T cells were seeded in the upper chamber (8 mm pore diameter; Corning Costar) in 100 µl the same medium to coculture with target tumor cells which was along with hsBCL9_CT_-24 (5 uM) or vehicle in lower chamber. To exclude the effect of tumor cells, a blank lower chamber was added only with the same medium but without tumor cells. After 24 h, we counted the number of the CAR-T cells in the lower chamber that might migrated through the pore. Each experiment was repeated.

### Animal Experiments

Female NSG mice aged at 5–6 weeks were purchased from Shanghai Model Organisms Center, Inc. and kept in the specific pathogen-free(SPF) conditions (22 ± 1°C, 12/12 light/dark cycle) in Fudan University for *in vivo* studies. After their adaption, every mouse was subcutaneously injected HCT116 cells harvested from cell culture and resuspended in PBS at a concentration of 5 × 10^6^ cells/100 µl on the right flank. When the growing tumors below the skin surface reach a palpable size, mice were randomly assigned. First, to verify the killing efficacy of CAR-T cells modified with EpCAM CAR against the human colorectal cancer cells expressed specific antigen of EpCAM *in vivo*, mice were randomized into three groups on day 5 and treated as following: untreated (PBS), control/mGFP CAR T cells and EpCAM CAR T cells (8 × 10^6^ cells, by i.v. every other day for 3 times). Then to investigate whether EpCAM CAR T cells combined with hsBCL9_CT_-24 could act synergistically for human colorectal cancer xenografts, mice were randomized into five groups before treatment on day 6 and treated as following: untreated/vehicle alone (2.5% DMSO in 5% Glucose), control/mGFP CAR T cells(8 × 10^6^ cells, by i.v., every other day for 4 times), EpCAM CAR T cells(8 × 10^6^ cells, by i.v., every other day for 4 times), hsBCL9_CT_-24 peptide(15 mg/kg, by i.p., with daily dose for 14 days),and EpCAM CAR T cells synergized with hsBCL9_CT_-24 peptide. A digital caliper was used to measure the length and width of the tumor, and the following formula was applied to calculate the volume of the tumor: tumor volume = length × width^2^/2. At the end of the experiment, the mice were sacrificed for other tests. During the experiments, mice were routinely monitored about their mobility, eye/hair matting, food and water consumption, bodyweight gain/loss, and other abnormal effects of tumor growth and treatments. Animal study followed the ARRIVE guidelines for the design, execution, analysis, and reporting of scientific research. All the procedures were performed in keeping with regulations of the Association for Assessment and Accreditation of Laboratory Animal Care International. It was reviewed and approved by School of Pharmacy in Fudan University of ethics committee.

### Chemicals and Peptide

hsBCL9_CT_-24 was produced by AnaSpec, CA, according to previous protocols (Ganesh et al., 2018)^.^ Analytical high-performance liquid chromatography (HPLC) and mass spectrometry (MS) were applied to evaluate its synthesis and purification. It was dissolved into 10 mmol/L and diluted before assay. Ac-LQTLRXIQRXL-2-Nal-NH2. X indicates (S)-2-(4′-pentenyl) Ala, B denotes norleucine (substituted for methionine to optimize activity of the ruthenium catalyst), 2- Nal represents 2-naphthylalanine, and β-Ala indicates β-alanine.

### The Staining of Hematoxylin-Eosin and Immunohistochemical

To evaluate acute toxic effects induced by hsBCL9 _CT_-24 or CAR-T cell, all treated mice were sacrificed and the sections of main visceral tissues were fixed by formalin and then embedded by paraffin. After further sections and deparaffinization, the slides were stained with H&E for morphologic analysis. For detecting infiltration of T cells in tumors, tumor tissues were fixed following euthanasia. Sections were prepared for paraffin, followed by deparaffinization and rehydration. After blocking, slides were incubated with the primary Abs: CD3ε (D7A6E™) XP^®^ Rabbit mAb (1:200, Cell Signaling Technology, #85061, United States) overnight at 4°C, followed by incubation with HRP-conjugated secondary Abs EnVision+/HRP Rabbit Ab (Dako 4003, Lot 10069185) for 30 min at room temperature. Then through color development with DAB Chromogen (Dako), counterstaining with Harris hematoxylin (Leica), differentiation with HCl-alcohol (70%) for 1–2 s, dehydration, finally the section was visualized (Ultra Vision Quan to Detection System).

### Statistical Analysis

The statistical analyses were performed by GraphPad Prism 7. Statistical differences between the results of two groups were evaluated using a two-tailed Student’s t-test, and multiple corrections using One-way ANOVA. The differences with *p* < 0.05 were considered statistically significant and symbols indicate statistical differences as follow *p < 0.05; **p < 0.01; ***p < 0.001, ****p < 0.0001).

## Results

### EpCAM Expression Associated with Metastasis and Survival in Cancer

EpCAM is an antigen expressed on most normal epithelial cells as well as in gastrointestinal carcinomas (Trzpis et al., 2007). EpCAM is expressed in a variety of human epithelial tissues and progenitor and stem cells. However, EpCAM is not found in non-epithelial cells or cancers of non-epithelial origin (Momburg et al., 1987). Our results showed that EpCAM has relatively higher expression in small intestine, colon, and thyroid, while EpCAM expression is less in other tissues (Supplementary Figure S1A). EpCAM in colon cancer shows a much higher number of transcripts per million than in normal colon tissue (Supplementary Figure S1B). EpCAM also shows significantly higher expression in multiple cancers than in normal tissue, for example in head-neck squamous cell carcinoma (HNSC), squamous-cell skin cancer (CESC), bladder cancer (BLCA), breast carcinoma (BRCA), colon cancer (COAD), esophageal carcinoma (ESCA), lung adenocarcinoma (LUAD), lung squamous cell carcinoma (LUSC), ovarian cancer (OV), pancreatic adenocarcinoma (PAAD), prostate adenocarcinoma (PRAD), rectal cancer (READ), stomach adenocarcinoma (STAD), tenosynovial giant cell tumor (TGCT), thymic carcinoma (THYM), uterine corpus endometrial carcinoma (UCEC), and uterine carcinosarcoma (UCS) (Supplementary Figures S1C, S1D).

**FIGURE 1:**
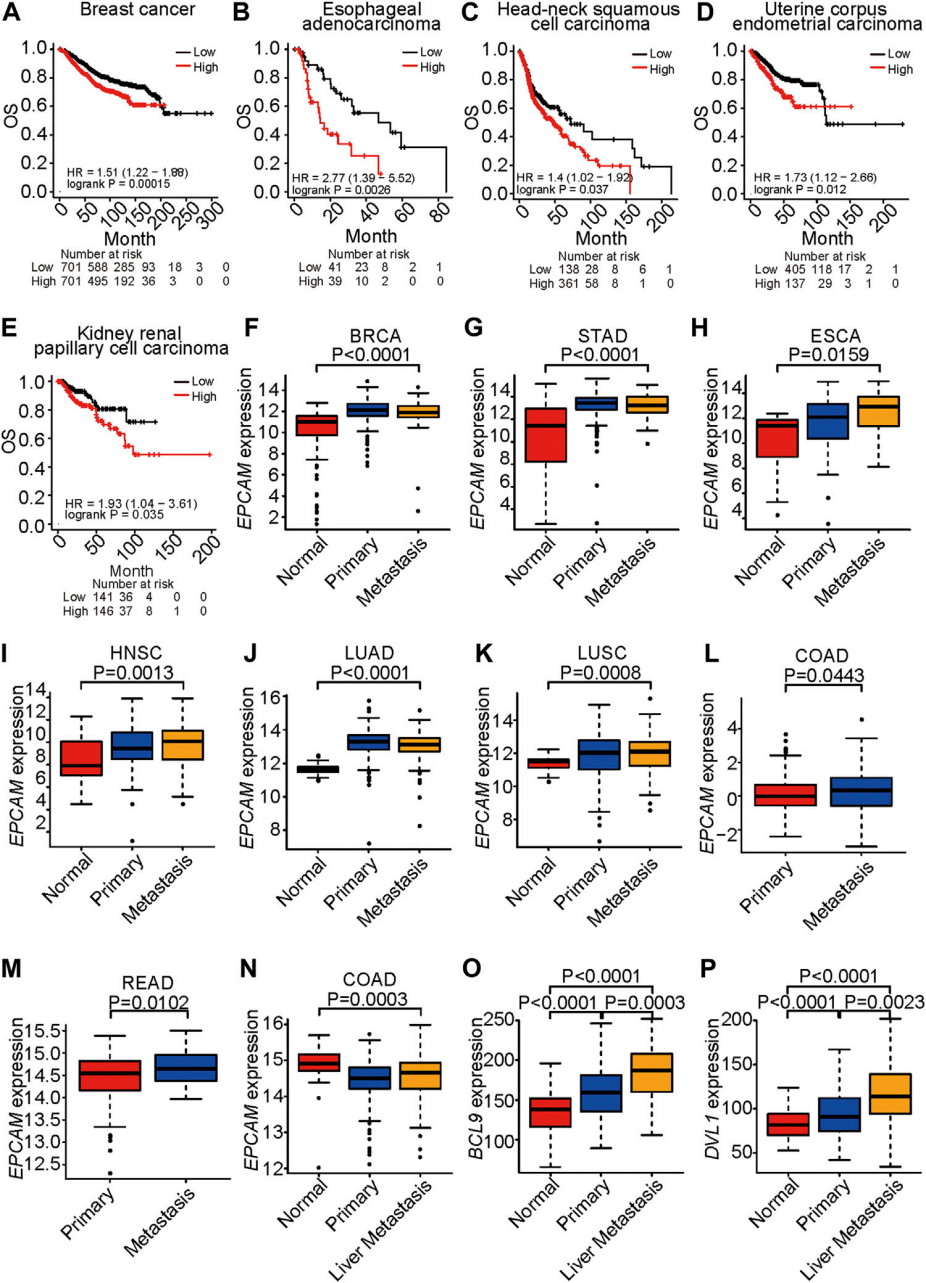
*EPCAM* expression is correlated to survival and metastasis. **(A–E)**, Survival analysis of TCGA patients with breast cancer **(A)**, esophageal adenocarcinoma **(B)**, head-neck squamous cell carcinoma **(C)**, uterine corpus endometrial carcinoma **(D)** and kidney renal papillary cell carcinoma **(E)**, respectively. OS represents overall survival. Classification is according to expression level of *EPCAM*. **(F–K)**, *EPCAM* expression level in normal, primary and metastatic tissue in BRCA **(F)**, STAD **(G)**, ESCA **(H)**, HNSC **(I)**, LUAD **(J)**, LUSC **(K)**, respectively. *EPCAM* expression level in primary and metastatic tissue in COAD **(L)**, READ **(M)**. **(N–P)**, *EpCAM* **(N)**, *BCL9* **(O)**, *DVL1* **(P)** expression level, respectively, in normal, primary and liver metastatic tissue in colorectal cancer.

EpCAM has been reported to play an important role in cancer development (Schmidt et al., 2008). In an analysis of patients with ovarian cancer, lung cancer, or stomach cancer, we found that those with high expression of EpCAM shown significantly shorter survival times, suggesting that EpCAM plays a part in the development of breast cancer, ESCA, HNSC, LUAD, LUSC, and stomach adenocarcinoma (Figures 1A–E).

EpCAM is an important marker of the cancer stem cells that are the major cause of metastasis (Zhang et al., 2020). An analysis of TCGA datasets indicated that expression of EpCAM was significantly increased in metastatic samples compared with normal tissues in BRCA, STAD, ESCA, HNSC, LUAD, and LUSC (Figures 1F–K). Expression of EpCAM was significantly increased in metastatic samples compared with primary tumor tissues in COAD and READ (Figures 1L–M). The liver is the most common site for metastases of CRC, breast cancer, and lung cancer. In an analysis of a GEO dataset, expression levels of *EpCAM*, *BCL9,* and *DVL1* were increased in colon cancer liver metastasis compared with primary tumors (Figures 1N–P). In these results, *EpCAM* shows significantly increased expression in the metastatic stage of cancer compared with primary tumors, indicating that EpCAM is a potential therapeutic target for metastatic cancer.

These results suggest that EpCAM shows increased expression in cancer cells compared with normal cells and that it is associated with cancer progression and metastasis.

### EpCAM is Correlated with Wnt Signaling in Cancer

**FIGURE 2:**
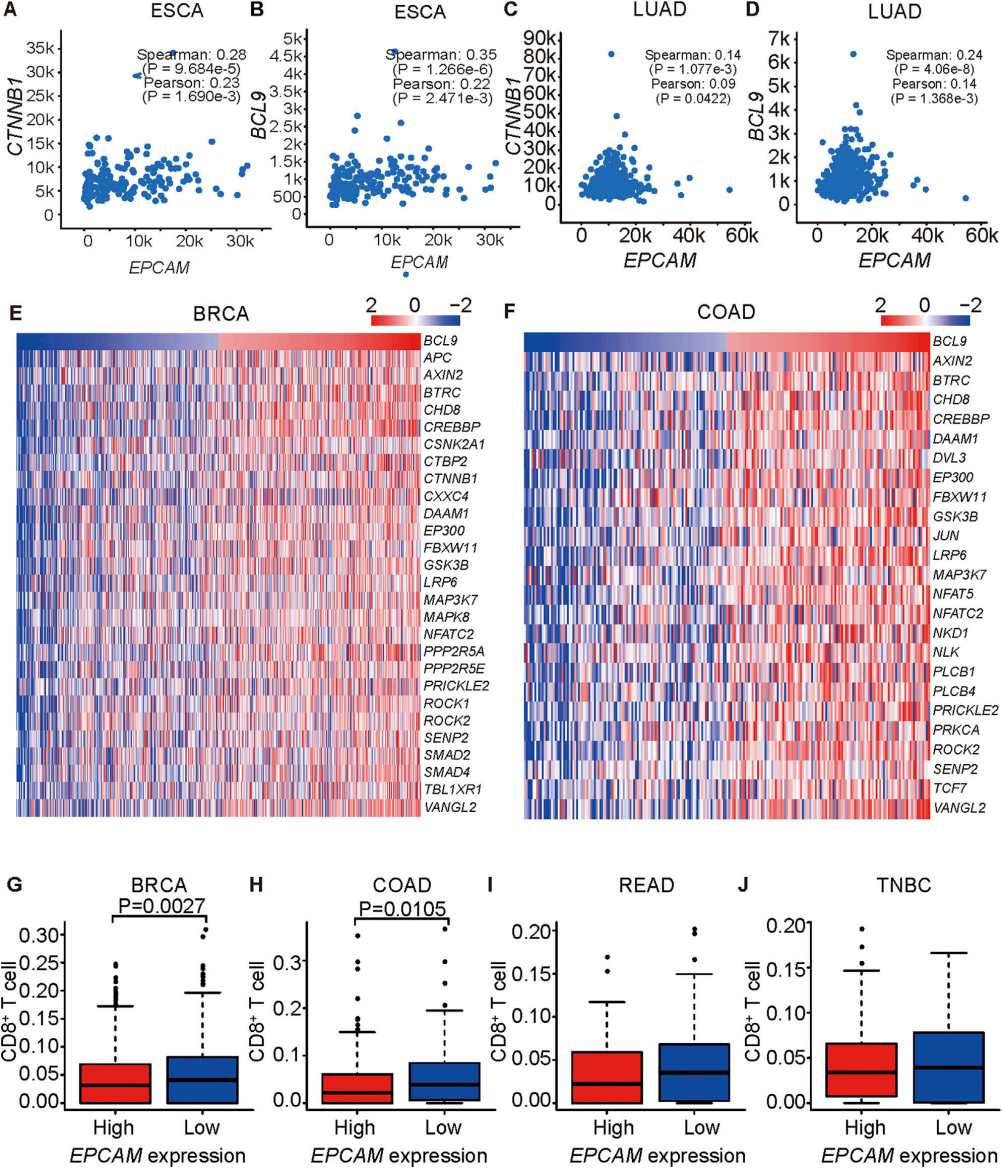
*EPCAM* expression is correlated to *BCL9* and *CTNNB1* expression. **(A–H)** Correlation analysis of *EPCAM* and *BCL9* expression level, *EPCAM* and *CTNNB1* expression level in ESCA **(A, B)**, LUAD **(C, D)**. **(E–F)** TGF β signaling pathway genes are correlated with BCL9 in BRCA **(E)**, COAD **(F)** of TCGA. **(G–J)** CD8+ T cells are associated with *EPCAM* expression level in BRCA **(G)**, COAD **(H)**, READ **(I)**, TNBC **(J)** of TCGA.

To validate whether a correlation of *EpCAM* and *BCL9*/*CTNNB1* exists in cancers, we analyzed the correlations of *EpCAM* and *BCL9* as well as *EpCAM* and *CTNNB1* in lung cancer, colon cancer, esophageal cancer, stomach cancer, head and neck cancer, and kidney cancer. *EpCAM* expression was positively correlated with the expression of *CTNNB1* in LUAD, COAD, ESCA, LUSC, and stomach cancer (Figures 2A,C and Supplementary Figures S2A–S2C). *EpCAM* expression was positively correlated with the expression of *BCL9* in LUAD, ESCA, HNSC, and kidney renal papillary cell carcinoma (KIRP) (Figures 2B,D and Supplementary Figures S2D, S2E).

Expression levels of *BCL9* and genes in the TGF *β* pathway were positively correlated in the BRCA and COAD patient samples (Figures 2E,F). The TGF *β* pathway is important in immune resistance (Pickup et al., 2013). Wnt/β-catenin was reported to inhibit T cell infiltration, and EpCAM depletion led to a decrease of Wnt/β-catenin expression (Yamashita et al., 2007; Spranger et al., 2015), so we explored the relationship between EpCAM expression and CD8^+^ T cells infiltration in cancers by a CIBERSORT analysis of tissues, and we found that they were inversely correlated (Figures 2G–J). These results suggested a positive role of EpCAM in immune resistance in cancers.

### Generation and Validation of EpCAM CAR T Cells

**FIGURE 3:**
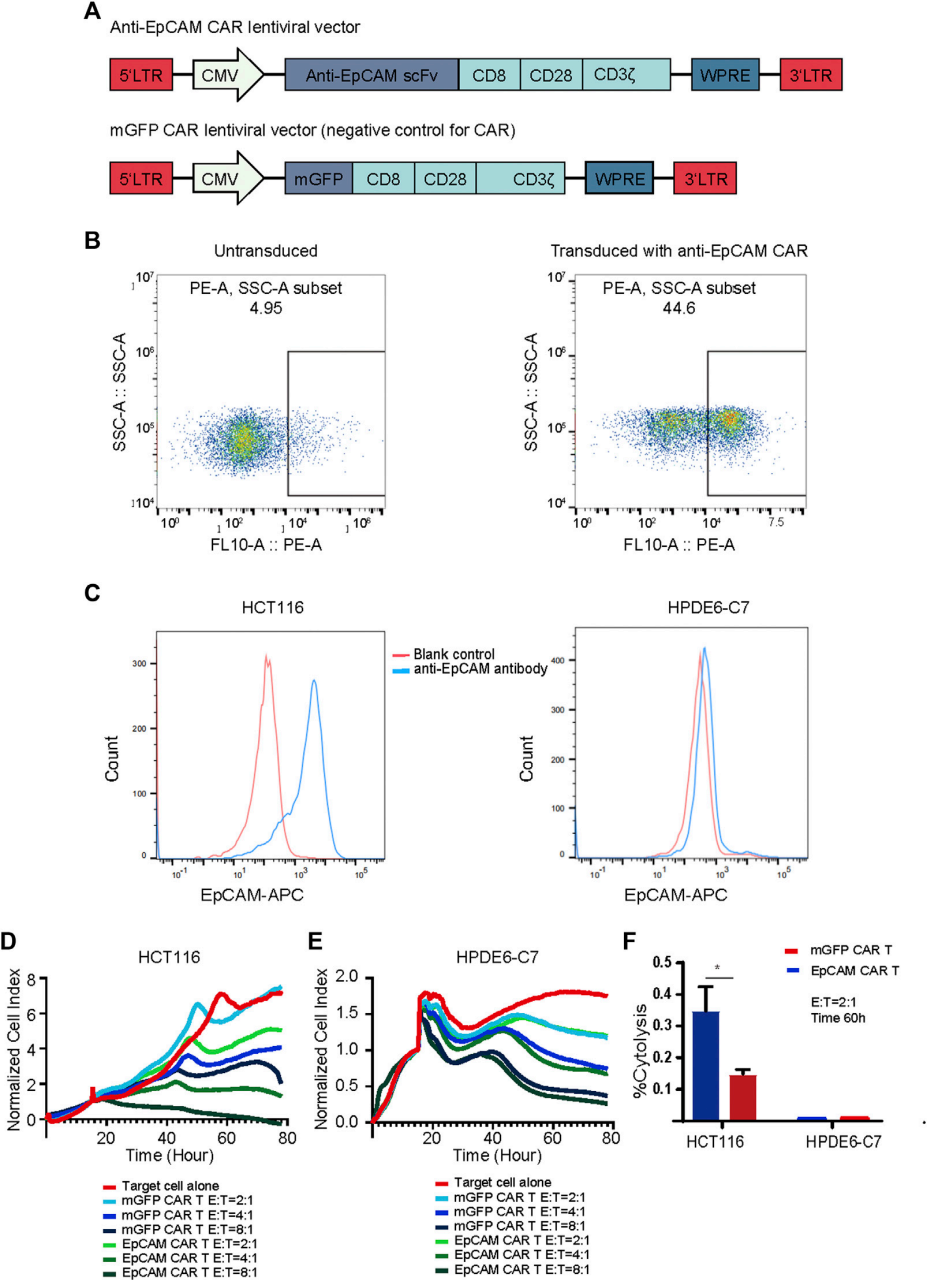
Generation and validation of EpCAM CAR T cells. **(A)** Schematics of lentiviral vectors used in the study. **(B)** Flow cytometric analysis of the surface expression of anti-EpCAM CAR on the modified T cells using recombinant biotinylated protein L to bind to scFv after 48 h of transduction. **(C)** Flow cytometric analysis of the surface expression of EpCAM on HCT116 and HPDE6-C7 using APC conjugated anti-human CD326 (EpCAM) antibody. The red line indicates the blank control while the blue line indicates the cells stained with anti-EpCAM antibody **(D)** RTCA application of EpCAM CAR T cells against EpCAM-high expressed HCT116 cancer cells. As target adherent cancer cells, HCT116 cells were seeded at a density of 5000 cells/well in a 16- well E-Plate and then moved to the incubator until they reached a logarithmic growth phase. mGFP-CART, EpCAM CAR T cells were added into the plate at E: T ratio of 8:1, 4:1 and 2:1, in duplicate, and the proliferation of the HCT116 cells was continuously monitored, reflected by cell index values (CI). Y-axis is the normalized cell index produced by the RTCA software (samples have been internally normalized for the Cell Index value measured before T cells addition) and X-axis is the time of cell culture and treatment time in hour; **(E)** RTCA application of EpCAM CAR T cells against EpCAM-low expressed HPDE6-C7 normal cells; **(F)** %Cytolysis calculated based on the analysis of RTCA of **(D)** and **(E)**, at 60 h and E:T ratio of 2:1. Each experiment was performed in duplicate at least.

To generate EpCAM CAR T, we constructed a lentiviral transfer plasmid encoding a second-generation CAR consisting of an anti-EpCAM single chain variable fragment (scFv), a CD8 hinge and transmembrane domain, a CD28 co-stimulatory domain, and a CD3ζ in intracellular domain (Figure 3A). The control lentiviral vector was constructed with the green fluorescent protein (GFP) coding sequence replacing the anti-EpCAM scFv sequence and was named membrane-bound GFP (mGFP) CAR. The viruses encoding the anti-EpCAM CAR or mGFP CAR were respectively transduced to human PBMCs that had been stimulated by anti-human CD3/CD28 immune magnetic beads for 4 days. To examine the transduction efficiency, we used recombinant biotinylated protein L binding to scFv and PE-conjugated streptavidin to detect the expression of anti-EpCAM CAR on transduced PBMCs. As shown in Figure 3B, after 48 h of transduction, 30–40% of PBMCs expressed the CAR. Then, based on the positive expression of EpCAM on HCT116 by flow cytometry (Figure 3C), RTCA was preliminarily performed to validate the EpCAM CAR T *in vitro*. The results showed that EpCAM CAR T cells had stronger killing activity against HCT116 at E:T ratios of 8:1, 4:1 and 2:1 compared with the negative control T cells (Figure 3D), indirectly confirming the expression of EpCAM CAR in modified T cells.

### EpCAM CAR T Cells Elicited Specific Cytotoxicity Against Target Cells Expressing the EpCAM Antigen

EpCAM is also reported to be expressed in normal cells, and thus we investigated whether EpCAM CAR T could show selective cytotoxicity to cancer cells compared with normal cells. HPDE6-C7 cells were developed from normal pancreatic duct epithelial cells that are the putative cells of origin of pancreatic ductal adenocarcinoma. There was no significantly different cytotoxicity against HPDE6-C7 between EpCAM CAR T and mGFP CAR T, and EpCAM CAR T showed good selectivity in inhibiting HCT116 cancer cell growth compared with HPDE6-C7 in RTCA (Figures 3D–F).

**FIGURE 4:**
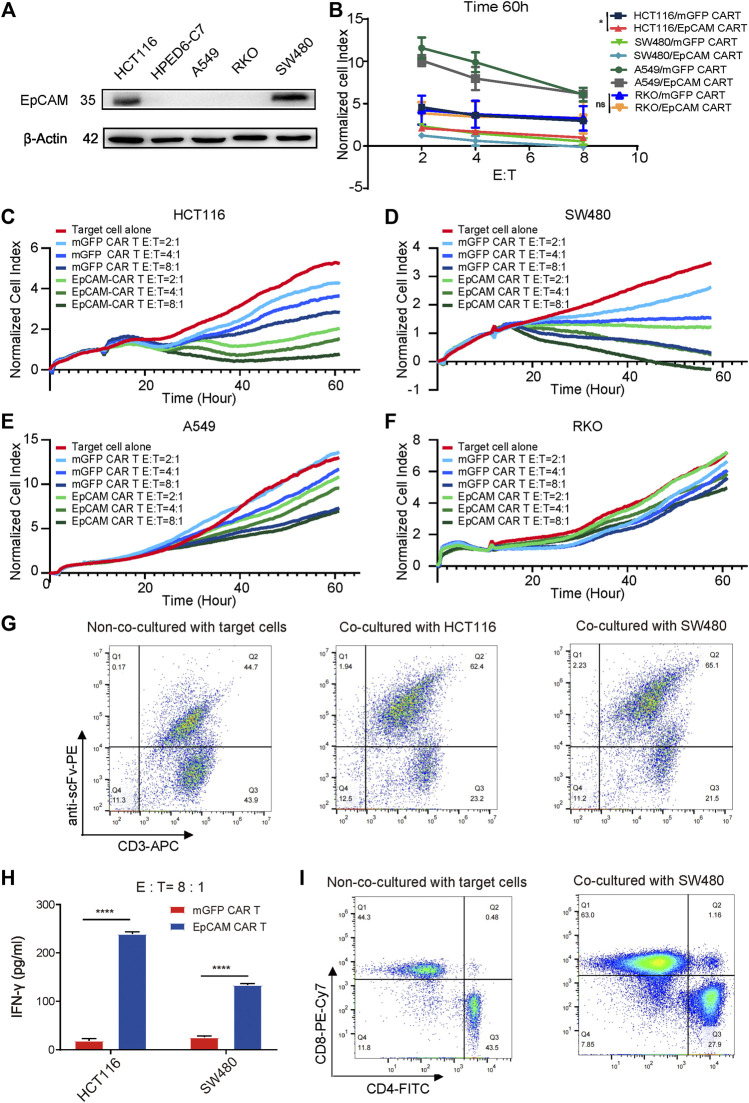
The specific cytotoxicity of EpCAM CAR T against target cells expressing EpCAM antigen. **(A)** WB was used to detect EpCAM expression on a set of cells. **(B)** The quantitative analysis of RTCA at time of 60 h of HCT116, SW480, A549 and RKO. The selective cytotoxicity of EpCAM CAR T was analyzed by RTCA, performed as above. Here the different cell lines HCT116 **(C)**, SW480 **(D)**, A549 **(E)**, and RKO **(F)** were taken as target cells respectively and the effector cells were added at different E: T of 2:1, 4:1, 8:1, in duplicate. **(G)** The enrichment of EpCAM CAR T cells after 48 h of co-culture with HCT116 or SW480 cells analyzed by flow cytometric analysis of the percentage of EpCAM CAR T in CD3+ T cells using anti-CD3 antibody and recombinant biotinylated protein L binding to scFv. **(H)** The release of cytokine was analyzed by ELISA. The CAR-T cells were co-cultured with target cells at E: T ratio of 8:1 for 48 h and then the released IFN-γ in the supernatant were detected by ELISA. **(I)** The phenotype changes on transduced T cells after 48 h of coculture with SW480 cells assessed by flow cytometry staining for CD3, CD4 and CD8. Results are shown as means ± SEM for experiments performed in duplicate at least. The statistical significance of differences between groups was determined by the unpaired Student’s t test. *p < 0.05; **p < 0.01, ***p < 0.001.

To investigate the specific cytotoxicity of the EpCAM CAR T cells, the surface expression levels of EpCAM on HCT116, SW480, A549, and RKO were detected using a WB assay. EpCAM was highly expressed on HCT116 and SW480 but was low expressed or absent on A549 and RKO (Figure 4A), which was consistent with the result of detection by flow cytometry analysis (Supplementary Figure S3). RTCA was then performed to assess whether the CAR T cells could specifically recognize and kill EpCAM-positive cells. Compared with EpCAM-negative cell lines, A549 and RKO, EpCAM CAR T cells displayed stronger killing activity against EpCAM-positive cell lines HCT116 and SW480 at E:T ratios of 8:1, 4:1, and 2:1, respectively (Figure 4B). There was a significant difference in cytotoxicity between EpCAM CAR T cells and mGFP CAR T cells against EpCAM-positive cell lines HCT116 and SW480 (Figures 4C,D), whereas the difference was not significant or was slightly significant against EpCAM-negative cells A549 and RKO (Figures 4E,F). Additionally, the killing efficacy of EpCAM CAR T cells against EpCAM-positive colorectal cancer cells was positively correlated with the E:T ratio. These results further demonstrated a higher specificity of the EpCAM CAR T cells regarding the recognition and killing of EpCAM-positive target cells.

To reveal the effect of stimulation of EpCAM antigen on the enrichment of anti-EpCAM expressing T cells, the percentage changes of EpCAM CAR T cells were measured by flow cytometry staining with anti-human CD3 antibodies and recombinant biotinylated protein L binding to scFv. The changes were 44.7% without co-culturing with the target cells, increasing to 62.4% after co-culturing with HCT116 and 65.1% with SW480, respectively, for 48 h at an E:T ratio of 2:1 (Figure 4G), indicating that the EpCAM CAR T cells could expand and enrich in a CAR-mediated manner during the killing of EpCAM-positive target cells.

To evaluate the antitumor capacity of cytokines, IFN-γ secretion was determined by ELISA. Th-1 cytokine IFN-γ secretion is associated with antitumor activity of T cells for adoptive immunotherapy. CAR T cells were co-cultured with HCT116 or SW480 for 48 h at an E:T ratio of 8:1. After the incubation, the culture cell-free supernatant was collected, and levels of cytokine IFN-γ released by effector cells were detected by ELISA. The results showed significant elevation of IFN-γ in EpCAM CAR T cells treated via co-culturing compared with mGFP CAR T cells (Figure 4H). This supported the suggestion that EpCAM CAR T cells had stronger killing ability against EpCAM-expressing cancer cells than control T cells.

The phenotypic changes of transduced T cells were examined by flow cytometry staining with anti-CD3, anti-CD8, and anti-CD4 antibodies. The percentage of CD8+ T cells increased and was significantly greater than CD4+ T cells after co-culturing with EpCAM-positive cancer SW480 cells for 48 h. Among CD3+ T cells, 44.3% were CD8+ and 43.5% were CD4^+^ T cells without co-culturing with target tumor cells, while 63% were CD8^+^ and 27.9% were CD4+ T cells after co-culturing with SW480 (Figure 4I), suggesting that CD8+ T cells may play a more important role in specific cytotoxicity.

**FIGURE 5:**
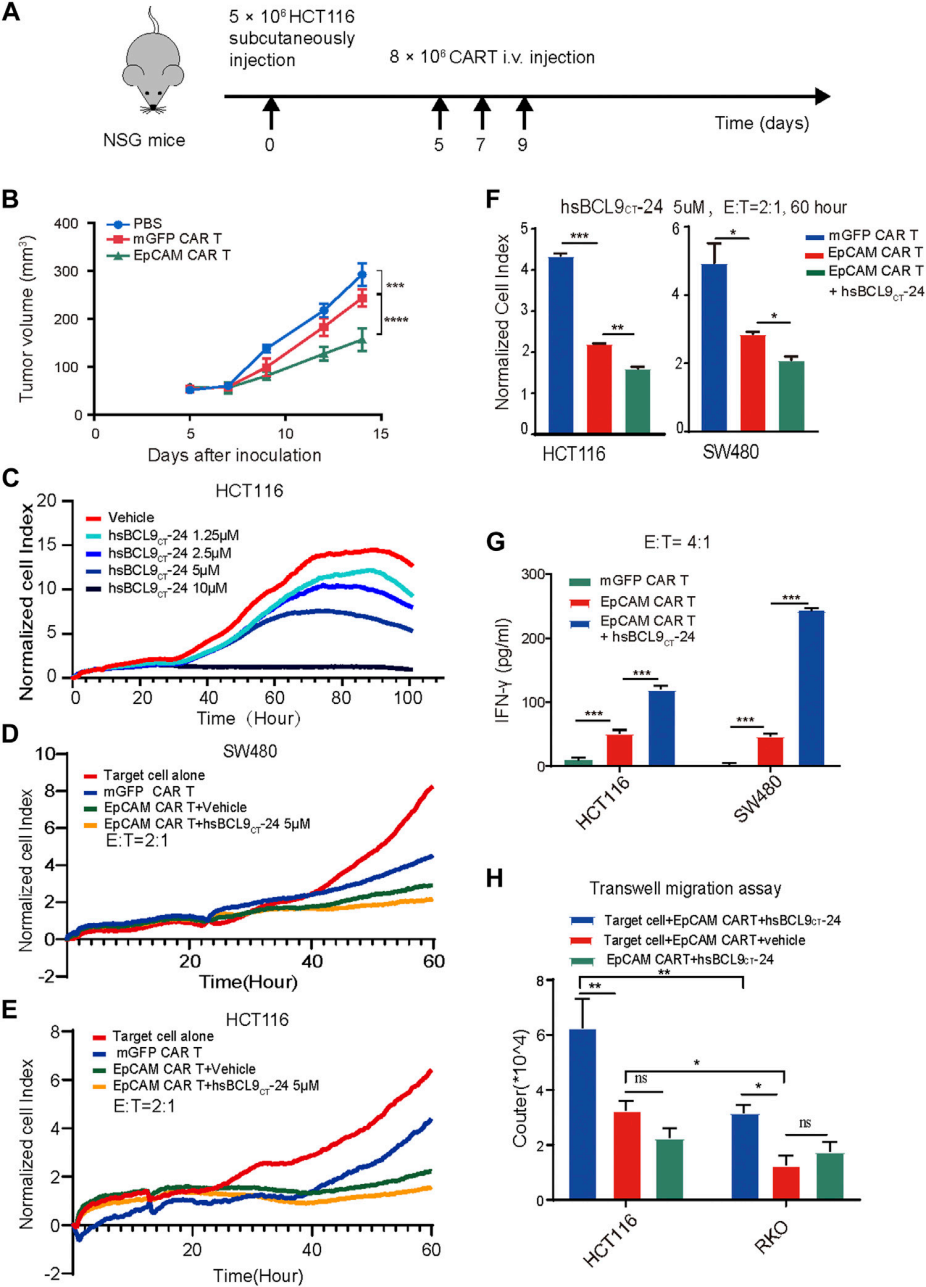
The specific tumor killing effect of EpCAM CAR T cells in HCT116 mouse model and synergetic activity of EpCAM CAR T and hsBCL9_CT_-24 *in vitro*. A total of 5 ×10^6^ HCT116 cells were subcutaneously injected into NSG mice on day 0 then randomized on day 5 and treated as following: untreated (PBS), mGFP CAR T cells and EpCAM CAR T cells (8 × 10 ^6^ cells, by i.v. every other day for 3 times). **(A)** Schematic diagram showing the *in vivo* experiment. The tumor growth curves during the experiment. **(C)** Effect of hsBCL9_CT_-24 alone on colorectal cancer cell was analyzed by dose-dependent RTCA, performed as above. Here was adding different concentration of hsBCL9_CT_-24, 1.25 µM, 2.5 µM, 5 µM, 10 µM instead of adding T cells. Synergetic activity of hsBCL9_CT_-24 with EpCAM CAR T on the colorectal cancer cells SW480 **(D)** or HCT116 **(E)** was detected by RTCA, performed as above, but the addition was mGFP CAR T, EpCAM CAR T, EpCAM CAR T+ hsBCL9_CT_-24 at E: T ratio of 2:1 and hsBCL9_CT_-24 at concentration of 5 µM, in duplicate. **(F)** The quantitative analysis of RTCA for synergeticactivity of hsBCL9_CT_-24 with EpCAM CAR Ton SW480 and HCt116 at E: T ratio of 2:1 and time of 60 h **(G)** The release of cytokine was analyzed by ELISA. The CAR-T cells were co-cultured with target cells at E: T ratio of 4:1 for 48 h treated with vehicle or hsBCL9_CT_-24 (5 µM) and then the released IFN_-γ_ in the supernatant were detected by ELISA. **(H)** Effect of hsBCL9_CT_-24 on migration of EpCAM CAR-T cells was analyzed by transwell migration assay. CAR-T cells were seeded in the upper chambers and cocultured with HCT116 or RKO cells seeded in the lower chambers pretreated with or without hsBCL9_CT_-24 (5 µM). After 24 h of co-culture, the number of the CAR-T cells that had migrated into the lower chamber was counted. Results are shown as means ± SEM for experiments performed in duplicate at least, and the statistical significance of differences between groups was determined by the unpaired Student’s t test. *p < 0.05; **p < 0.01, ***p < 0.001.

To further test the specific tumor killing effects of EpCAM CAR T cells *in vivo*, the colon tumor cell line HCT116 with positive expression of EpCAM was selected to establish a xenograft tumor model. A schematic of the experiment is shown in Figure 5A. A total of 5 × 10^6^ HCT116 cells were subcutaneously injected into NSG mice on day 0. On day 5 when tumors grew to a palpable size, the mice were randomly assigned to the following three regimens: untreated (PBS), mGFP CAR T/control T cells, and EpCAM CAR T cells (8 × 10^6^ cells by i.v. every other day for three times). As shown in Figure 5B, the volumes of tumors in the untreated and control T groups progressively increased from day 7 to 14, although the tumors of the control T group grew significantly slower than those of the untreated group. The volumes of tumors in the EpCAM CAR T group remained low, showing stronger suppression of tumor growth than in the control T group. This result was further evidence that the EpCAM CAR T cells had a specific killing effect against HCT116 tumors.

These results all demonstrated that the EpCAM CAR T cells exhibited a stronger response to the specific antigen *in vitro* and *in vivo*, especially the CD8+ T cells.

### Synergetic Activity of hsBCL9_CT_-24 with EpCAM-CAR-T on Cancer Cells *In Vitro*

First, to evaluate the effect of hsBCL9_CT_-24 alone on colorectal cancer cells, we conducted dose- dependent RTCA. The CI-t curves of HCT116 or SW480 cell lines based on different concentration of hsBCL9_CT_-24, 1.25 µM, 2.5 µM, 5 µM, and 10 µM, showed gradient degrees of decreases in CI; 10 µM had the strongest effect, demonstrating that hsBCL9_CT_-24 could suppress the proliferation of HCT116 or SW480 cells in a dose-dependent manner (Figure 5C). We further measured the cytotoxicity of a combination of hsBCL9_CT_-24 and CAR T cells on HCT116 and SW480 cells by RTCA (hsBCL9_CT_-24 5 µM, E: T=4). The results showed a significant decline of CI in the combination-treated target cells compared with the EpCAM CAR T treatment alone, suggesting that hsBCL9_CT_-24 treatment could ameliorate the cell-mediated cytotoxicity of EpCAM CAR T cells *in vitro* (Figures 5D–F).

In cytokine release assays, the released IFN-γ in the supernatant was detected by ELISA after the same co-culture. The results showed that levels of the cytokine IFN-γ released by a combination of EpCAM CAR T cells and hsBCL9_CT_-24 was significantly higher than by vehicle- treated EpCAM CAR T cells (Figure 5G), indicating that hsBCL9_CT_-24 could enhance the antitumor potential of EpCAM CAR T cells.

In addition, a transwell migration assay was performed to investigate whether hsBCL9_CT_-24 could affect the migration of EpCAM CAR T cells. The lower chambers contained cell-free medium, target cells+ vehicle, and target cells+ hsBCL9_CT_-24. The results showed that after 24 h at 37°C, the number of CAR T cells treated with target cells+ hsBCL9_CT_-24 that had migrated into the lower chamber was significantly greater than the others, suggesting that hsBCL9_CT_-24 could increase the migration of CAR T cells *in vitro* (Figure 5H). Meanwhile, the migrated CAR T cells in target cell HCT116 group was significantly greater than those in RKO group, which also indicating the antigen specific.

The above results indicated that hsBCL9_CT_-24 could promote the CAR T cells capacity for cytotoxicity and production of IFN-γ as well as migration toward the antigen.

### Synergy of hsBCL9_CT_-24 with EpCAM CAR T in Antitumor Activity *In Vivo*

**FIGURE 6:**
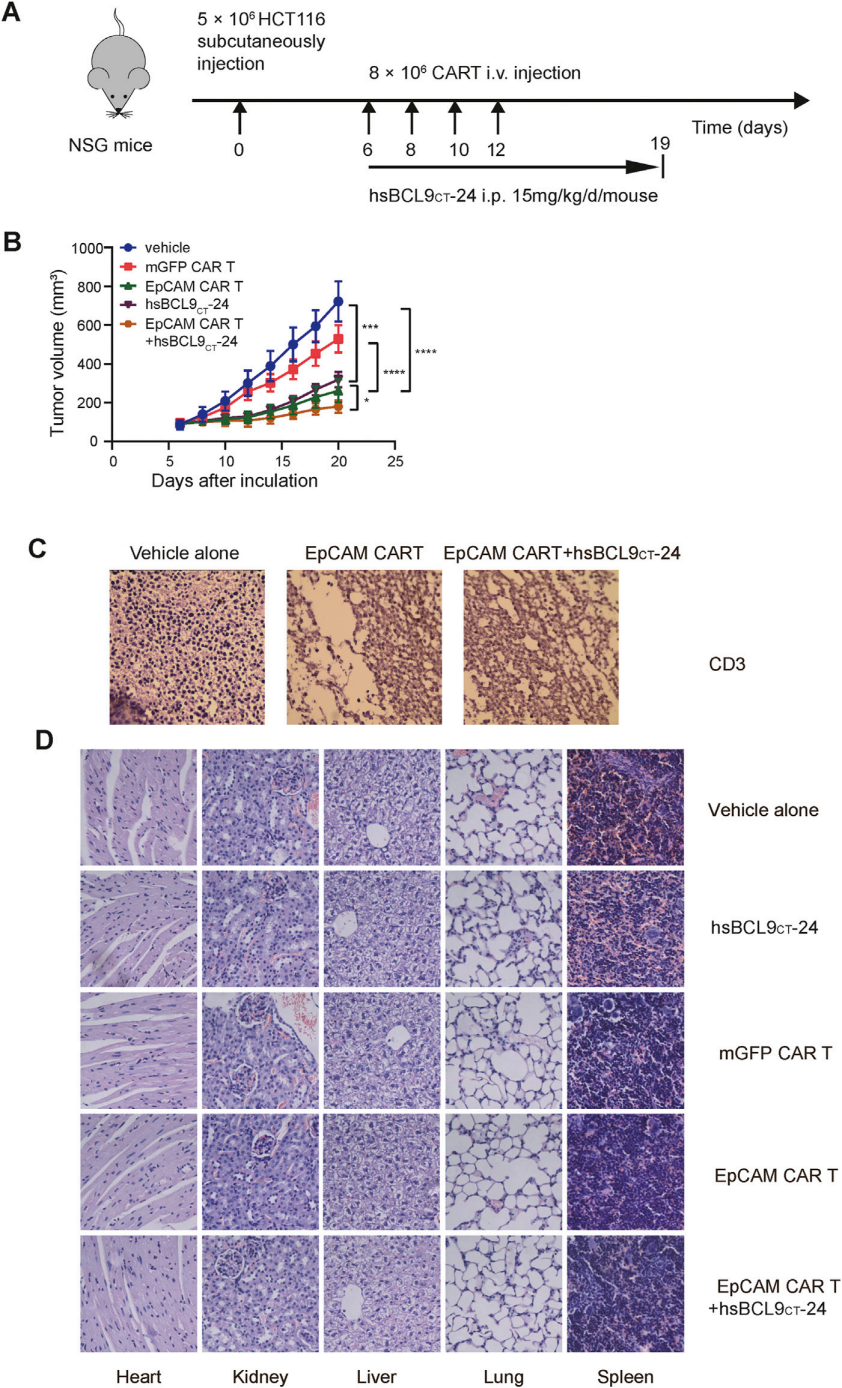
Synergetic activity of hsBCL9_CT_-24 with EpCAM CAR T in HCT116 mouse model and safety evaluation of CAR-T cells or hsBCL9-CT24 in the HCT116 tumor model. NSG mice were subcutaneously injected with 5 × 10^6^ HCT116 on day 0 then randomized on day 6 and treated as following: vehicle alone (2.5% DMSO in 5% Glucose), mGFP CAR T cells (8 × 106 cells, by i.v., every other day for 4 times), EpCAM CAR T cells (8 × 106 cells, by i.v., every other day for 4 times), hsBCL9_CT_-24 peptide (15 mg/kg, by i.p., with daily dose for 14 days), and EpCAM CAR T cells synergized with hsBCL9_CT_-24 peptide. **(A)** Schematic diagram showing the in vivo experiment. **(B)** The tumor growth curves during the experiment. **(C)** The infiltration of T cells into tumor by IHC staining for CD3ε. **(D)** H&E staining of main visceral tissues, including the heart, liver, kidney, lung and spleen from treatment mouse (400×). Results are shown as means ± SEM for experiments performed in triplicate, and the statistical significance of differences between groups was determined by the unpaired Student’s t test. *p < 0.05; **p < 0.01, ***p < 0.001.

We proceeded to evaluate the therapeutic activity of mGFP CAR T cells/control T cells, EpCAM CAR T cells, and EpCAM CAR T cells plus hsBCL9_CT_-24 in NSG mice with subcutaneous xenograft tumor models established with HCT116. A schematic is shown in Figure 6A. In accord with previous results for the specific tumor-killing effect of EpCAM-CAR T cells alone *in vivo*, the volumes of tumors in the EpCAM CAR T group increased significantly slower than those in the mGFP CAR T group. The key point here was that the EpCAM CAR T cells plus hsBCL9_CT_-24 significantly suppressed the growth rates of tumors compared with the EpCAM CAR T cells alone (Figure 6B). These results verified the combined antitumor efficacy of EpCAM CAR T cells and hsBCL9_CT_-24, and hsBCL9_CT_-24 could ameliorate the ability of EpCAM CAR T in cancer therapy. To directly assess the infiltration of T cells into tumors, IHC staining for CD3ε in freshly collected tumor tissues was performed; as shown in Figure 6C, the signal of CD3ε expression was up-regulated in tumor tissue of the combination treatment group compared with the group given CAR T cells alone, while the group without T cell treatment showed an absence of CD3ε expression. These results remained consistent with the above *in vitro* findings.

### Safety Evaluation of CAR T Cells and hsBCL9_CT_-24 in Mice

The potential toxic effects induced by hsBCL9_CT_-24 or CAR T cells in mice were evaluated *in vivo* experiments. During the entire treatment process, there were no graft-versus-host reactions such as diarrhea or rash in any of the mice. The H&E staining of main visceral tissues such as heart, kidneys, liver, lungs, and spleen from treated mice showed no obvious toxic pathologic changes (Figure 6D). These data supported the safety of application of CAR T cells or hsBCL9_CT_-24 *in vivo* in mice.

### hsBCL9_CT_-24 Treatment Ameliorated the Tumor Microenvironment and Improved Infiltration of T Cells

**FIGURE 7:**
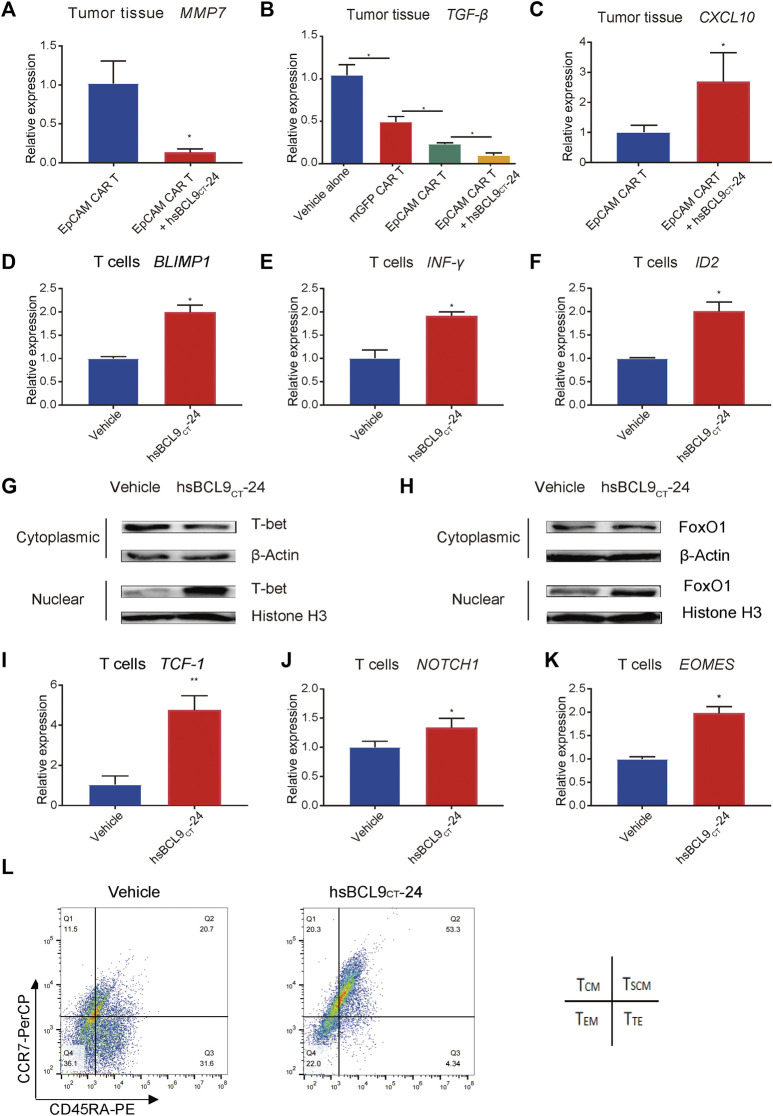
hsBCL9_CT_-24 treatment promoted molecules correlated with effector T cells and promoted the molecules correlated with early memory T cells. The total RNA of tumor tissues from mice was extracted using Trizol and qRT-PCR was performed. qRT-PCR measurement of **(A)** MMP7, **(B)** TGF-β, **(C)** CXCL10 expression. After 24 h of co-culture with HCT116 synergized with hsBCL9_CT_-24 or vehicle, the activated T cells were collected. qRT-PCR measurement of **(D)** BLIMP1, **(E)** IFN-γ, and **(F)** ID2 expression in the collected T cells. **(G)** T-bet in the nucleus and cytoplasm was detected by western blot. The nuclear and cytoplasmic proteins were extracted using the nuclear and cytoplasmic protein extraction kit. T-bet in the nucleus and cytoplasm was analyzed with an anti-T-bet antibody. Histone H3 in the nucleus and β-actin in the cytoplasm were also detected as internal controls. **(H)** FoxO1 in the nucleus and cytoplasm were detected by western blot. The nuclear and cytoplasmic proteins were extracted using the nuclear and cytoplasmic protein extraction kit. FoxO1 in the nucleus and cytoplasm was analyzed with an anti- FoxO1 antibody. Histone H3 in the nucleus and β-actin in the cytoplasm were also detected as internal controls. Over 48 h of co-culture with HCT116 synergized with hsBCL9_CT_-24 or vehicle, the activated T cells were collected. **(I–K)** qRT-PCR measurement of **(I)** TCF-1, **(J)** NOTCH1, and **(K)** EOMES expression in the collected T cells. **(L)** The memory phenotype of the CAR-T cells was analysed by flow cytometry with anti-CCR7 and anti-CD45RA antibodies after 48 h of co-culturing with HCT116 cells, adding hsBCL9_CT_-24 or vehicle. Results are shown as means ± SEM for experiments performed in triplicate, and the statistical significance of differences between groups was determined by the unpaired Student’s t test. *p < 0.05; **p < 0.01; ***p < 0.001.

Our previous data showed that hsBCL9_CT_-24 could affect tumor microenvironment, such as increasing dendritic cells and reducing regulatory T cells (Feng et al., 2019). In this study, we chose the following two factors to estimate the improvement of the tumor microenvironment by hsBCL9_CT_-24. Transforming growth factor β (TGF-β), as an immunosuppressive factor, shapes the tumor microenvironment to restrain anti-tumor immunity by restricting T-cell infiltration (Mariathasan et al., 2018). Among all known human chemokines, CXCL10 is known to be strongly associated with CD8^+^ T-cell infiltration (Romero et al., 2020). As shown in Figure 7A, after treatment with hsBCL9_CT_-24, MMP7, one of the Wnt/β-catenin signaling pathway targets, was significantly downregulated in tumor cells, as expected. Meanwhile, in analyses of TGF-β expression in tumor models *in vivo*, the combination of the EpCAM CAR T cells and hsBCL9_CT_-24 exhibited the lowest level, significantly reduced compared to the group of EpCAM CAR T cells alone without hsBCL9_CT_-24 treatment. This demonstrated that hsBCL9_CT_-24 treatment could further inhibit TGF-β expression in tumor tissues (Figure 7B). In contrast, the level of CXCL10 was significantly upregulated in the combined hsBCL9_CT_-24 treatment group compared with the EpCAM CAR T cells alone group (Figure 7C). These results indicated the positive effect of hsBCL9_CT_-24 on improving infiltration of T cells by potentially downregulating TGF-β and upregulating CXCL10 in tumor tissues.

### hsBCL9_CT_-24 Treatment Affected the Subsets of T Cells and Facilitated their Anti-Tumor Efficacy

To investigate the effect of hsBCL9_CT_-24 on T cells during the antitumor process, we focused on the subsets of changes and tested the expression levels of several molecules correlated with different subsets. The T-box transcription factor T-bet (T-box expressed in T cells) has been implicated as a master regulator of early effector CD8 T cells (Joshi et al., 2007), and it activates along with nuclear localization (McLane et al., 2013). In addition, T-bet promotes cytotoxic lymphocyte formation and upregulates IFN-γ expression. Besides T-bet, B lymphocyte-induced maturation protein-1 (Blimp1) (Kallies et al., 2009) and inhibitor of DNA binding protein 2 (ID2) (Masson et al., 2013) are also critical and are upregulated in seeding Teff differentiation. In addition, the expression of *IFN-γ*, *BLIMP1*, and *ID2* on CAR T cells synergized with hsBCL9_CT_-24 were all significantly upregulated compared with the vehicle controls (Figures 6D–F). These data suggested that hsBCL9_CT_-24 may promote the effector T cells ability to kill tumor cells and to produce cytokines at the early stages upon T-cell receptor (TCR) stimulation.

To test whether synergized hsBCL9_CT_-24 led to the nuclear accumulation of T-bet in activated T cells, we performed WB analyses of protein fractions isolated from the nucleus and cytoplasm. As shown in Figure 7G, after 24 h of co-culturing of EpCAM CAR T cells with HCT116 cells, hsBCL9_CT_-24 treatment caused a significant increase in T-bet in the nucleus and a significant decrease in T-bet in the cytoplasm, indicating the activation of T-bet. Forkhead box protein O1 (FoxO1) has also been identified as a key promoter for memory CD8+ T-cell differentiation (Hess Michelini et al., 2013). Meanwhile the nuclear localization of FoxO1 in the CAR T cells synergized with hsBCL9_CT_-24 indicated greater activation (Figure 7H).

Over the course of 48 h following TCR stimulation, the following changes were found in the CAR T cells synergized with hsBCL9_CT_-24. First, the transcription factor T-cell factor 1 (TCF-1), encoded by Tcf7, is known as a downstream transcription factor of the canonical Wnt signaling pathway, but it is not exclusive. In other cases, TCF-1 is not regulated by canonical Wnt signals, and other upstream signals such as Notch1 could be required for TCF-1 regulation (Weber et al., 2011). TCF-1 is required for establishment of CD8 memory T cells in PBMC, while *β*-catenin is indispensable for CD8+ memory development (Prlic and Bevan, 2011). An analysis of CAR-T cells from treated CLL patients with complete disease remission showed an increase in memory CD8+ T-cell gene expression relative to non-responders to CAR T-cell therapy, and this included increased expression of TCF-1 (Fraietta et al., 2018). In addition, another T-box transcription factor, Eomes (Eomesodermin), is increasingly expressed as T cells progress from effector to memory cells (McLane et al., 2013). In our analysis of *TCF-1*, *NOTCH1*, and *EOMES* expression in T cells co-cultured with HCT116 cells, the CAR T cells synergized with hsBCL9_CT_-24 all had higher levels than those added the vehicle control (Figures 7I–K). Meanwhile, the memory phenotype of the CAR-T cells was analysed by flow cytometry with anti-CCR7 and anti-CD45RA antibodies after 48 h. As shown in Figure 7L, the percentage of the stem central memory T cells (TSCM, CCR7+ CD45RA+) plus the central memory T cells (TCM, CCR7+ CD45RA−) was more than 70% in the hsBCL9_CT_-24 synergized CAR-T cells; however, it was only about 30% in the vehicle-treated CAR-T cells. These results implied that hsBCL9_CT_-24 may lead to increased generation of memory T cells after a period of stimulation by TCR.

## Discussion

We successfully constructed the EpCAM CAR T and verified the combination synergy between Wnt inhibitor and CAR-T. Our research has paved the way for exploring new opportunities for safer and more efficient immunotherapy.

CAR-T therapy has recently emerged as a promising treatment for hematological malignances. Unfortunately, the potent, durable response to this immunotherapy happened in a small subset of patients, the majority had little response (Sotillo et al., 2015). In solid tumors, CAR T-cell infiltration is low, presenting a challenge that needs to be overcome. We also need to further modify CAR-T cells or use other therapies to enhance CAR-T-cell infiltration (Parente-Pereira et al., 2011). In this study we found that blocking the oncogenic Wnt pathway by hsBCL9_CT_-24 could upregulate chemokine CXCL10 and improve T-cell tumor infiltration in cancer models, which are consistent with a recent report that Wnt pathway activation is linked to primary resistance in immunotherapy (Grasso et al., 2018). TGF-β makes great contribution to the tumor microenviroment (TME) and tumor progression (Bachman and Park, 2005). Inhibition of TGF-β signaling *via* BCL9/β-catenin suggests an approach for dual regulation of the TME and checkpoint blockade (Feng et al., 2019). In fact, besides the tumor infiltration, Teff are suppressed by TME in recognition and clearance of tumor cells, and the following persistence (Pickup et al., 2013). As described above, EpCAM increased expression in cancer cells compared with normal cells and it is associated with cancer progression and metastasis. *EpCAM* also exhibited inversely correlated with CD8+ T cells infiltration in cancers. Hence targeting both EpCAM and Wnt/β-catenin can not only inhibit tumor growth but also prompt tumor infiltration without affecting T-cell survival and enhances the response to EpCAM CAR T, making it an ideal strategy to reactivate intra-tumoral Teff cells and transform a “cold” immune microenvironment into a “hot” one.

Recent studies have revealed two major problems associated with a low response or a relapse during CAR-T-cell therapy. The first is the loss of CAR target molecules on tumor cells (Ruella et al., 2016). The second is the reduced *in vivo* persistence of transferred CAR-T cells, mostly due to T-cell exhaustion and dysfunction by continuous stimulation from TCR and cytokines (Schietinger et al., 2016). The emerging results support the strategy that reversal of T-cell exhaustion and reprograming to early memory T cells in humans is ideal for improving the efficacy of antitumor immune therapy (Gargett et al., 2019). In this study, we found that hsBCL9_CT_-24 had the potential to promote effector T cells with high capacity for cytotoxicity and production of IFN-γ at the early stages and then memory T cells with persistence at the later stages of the stimulation of TCR. The transformation of the CAR T cells phenotype during the treatment *in vitro* provided a new approach to resolve the problem of exhaustion. But the differentiation of T cells is a complicated and dynamic process; regarding the different models and/or possible post-transcriptional regulation of those genes, more researches are needed to verify the effect of hsBCL9_CT_-24 on T-cell differentiation, especially *in vivo*. The detailed mechanisms of hsBCL9_CT_-24 function in CAR−T immunotherapy remain to be determined.

There is an increasing number of clinical trials recently registered on EpCAM CAR-T(e.g., NCT02915445, NCT04151186, NCT03013712, NCT03563326, ChiCTR2100047129, etc) and there is no clear efficacy reported yet. There is still no viable combination approach with EpCAM CAR-T to overcome the challenge of TIME in solid tumor.

EpCAM was reported at stem cell marker. Wnt signaling plays important role in cancer stem cells (Yamashita et al., 2007). EpCAM and Wnt signaling could form an interaction cycle in colon cancer in cancer stem cell, immune modulation, etc. The novelty in our research is to report the synergetic activity of hsBCL9_CT_-24 with EpCAM-CAR-T, which was not reported in other combinational therapies of CAR-T before.

In addition, although CAR-T immunotherapy is relatively safe, it can also have some side effects such as cytokine release syndrome (CRS), which induces adverse reactions, even leading to the death of patients. This is a problem that needs to be overcome in the future (Neelapu et al., 2018). In this regard, the future direction of research may be concerned with how to manipulate CAR T cells, allowing them to kill cancer cells at the right time, with reasonable release of related cytokines. In the event of adverse reactions, the cessation of CAR T-cell proliferation and cytokine release should proceed in a timely manner so that CAR-T cells can be maximized for efficacy while ensuring patient safety (Abreu et al., 2020). As another problem in the CAR-T treatment, the loss of CAR target antigen also received much attention. The rapid progress of biotechnology should further the development of CAR-T technology, and research will continue to improve the safety and effectiveness of CAR T cells, making them an effective anti-cancer weapon. Meanwhile, exploring better combination therapy is another way to improve their therapeutic efficacy and decrease their shortcomings.

## Data Availability

The datasets presented in this study can be found in online repositories. The names of the repository/repositories and accession number(s) can be found in the article/Supplementary Material.

## Supplementary Material

Supplementary Figure S1
*EPCAM* expression in normal tissues and cancers. **(A)**, *EPCAM* expression level in normal tissues. **(B)**, *EPCAM* expression level in different types of cancer. **(C)**, *EPCAM* expression level in tumor and matched normal tissue (**p* < 0.05). **(D)**, IHC staining EPCAM in tumor and matched normal tissue from patients with colorectal cancer or stomach cancer.

Supplementary Figure S2
*EPCAM* expression is correlated to *BCL9* and *CTNNB1* expression. *EPCAM* and *CTNNB1* expression level in COAD **(A)**, LUSC **(B)** and stomach cancer **(C)**. Correlation analysis of *EPCAM* and *BCL9* expression level, in HNSC **(D)**, and KIRP **(E)**.

Supplementary Figure S3 Expression of EpCAM on several cells by flow cytometric analysis. **(A)** The expression of EpCAM on a variety of cells (Colo320, RKO, A549, HPDE6-C7, HCT116, SW480) was detected by flow cytometric analysis.The red line indicates the blank control while the blue line indicates the cells stained with anti-EpCAM antibody. **(B)** The expression of EpCAM on HCT116 was detected after or without hsBCL9_CT_-24 treatment. The red line indicates cells without treatment while the blue line indicates cells with the treatment.

## References

[B1] Abreu T. R. Fonseca N. A. Gonçalves N. Moreira J. N. (2020). Current Challenges and Emerging Opportunities of CAR-T Cell Therapies. J. Control. Release 319, 246–261. 10.1016/j.jconrel.2019.12.047 31899268

[B2] Bachman K. E. Park B. H. (2005). Duel Nature of TGF-Beta Signaling: Tumor Suppressor vs. Tumor Promoter. Curr. Opin. Oncol. 17, 49–54. 10.1097/01.cco.0000143682.45316.ae 15608513

[B3] Beavis P. A. Slaney C. Y. Kershaw M. H. Gyorki D. Neeson P. J. Darcy P. K. (2016). Reprogramming the Tumor Microenvironment to Enhance Adoptive Cellular Therapy. Semin. Immunol. 28, 64–72. 10.1016/j.smim.2015.11.003 26611350

[B4] Bray F. Ferlay J. Soerjomataram I. Siegel R. L. Torre L. A. Jemal A. (2018). Global Cancer Statistics 2018: GLOBOCAN Estimates of Incidence and Mortality Worldwide for 36 Cancers in 185 Countries. CA Cancer J. Clin. 68, 394–424. 10.3322/caac.21492 30207593

[B5] Chmielewski M. Abken H. (2015). TRUCKs: the Fourth Generation of CARs. Expert Opin. Biol. Ther. 15, 1145–1154. 10.1517/14712598.2015.1046430 25985798

[B6] Eshhar Z. Waks T. Gross G. Schindler D. G. (1993). Specific Activation and Targeting of Cytotoxic Lymphocytes through Chimeric Single Chains Consisting of Antibody-Binding Domains and the Gamma or Zeta Subunits of the Immunoglobulin and T-Cell Receptors. Proc. Natl. Acad. Sci. U S A. 90, 720–724. 10.1073/pnas.90.2.720 8421711 PMC45737

[B7] Feng M. Jin J. Q. Xia L. Xiao T. Mei S. Wang X. (2019). Pharmacological Inhibition of β-catenin/BCL9 Interaction Overcomes Resistance to Immune Checkpoint Blockades by Modulating Treg Cells. Sci. Adv. 5, eaau5240. 10.1126/sciadv.aau5240 31086813 PMC6506245

[B8] Finney H. M. Lawson A. D. Bebbington C. R. Weir A. N. (1998). Chimeric Receptors Providing Both Primary and Costimulatory Signaling in T Cells from a Single Gene Product. J. Immunol. 161, 2791–2797. 9743337

[B9] Fraietta J. A. Lacey S. F. Orlando E. J. Pruteanu-Malinici I. Gohil M. Lundh S. (2018). Determinants of Response and Resistance to CD19 Chimeric Antigen Receptor (CAR) T Cell Therapy of Chronic Lymphocytic Leukemia. Nat. Med. 24, 563–571. 10.1038/s41591-018-0010-1 29713085 PMC6117613

[B10] Ganesh S. Shui X. Craig K. P. Park J. Wang W. Brown B. D. (2018). RNAi-Mediated β-Catenin Inhibition Promotes T Cell Infiltration and Antitumor Activity in Combination with Immune Checkpoint Blockade. Mol. Ther. 26, 2567–2579. 10.1016/j.ymthe.2018.09.005 30274786 PMC6225018

[B11] Gargett T. Truong N. Ebert L. M. Yu W. Brown M. P. (2019). Optimization of Manufacturing Conditions for Chimeric Antigen Receptor T Cells to Favor Cells with a central Memory Phenotype. Cytotherapy 21, 593–602. 10.1016/j.jcyt.2019.03.003 30975603

[B12] Goff S. L. Dudley M. E. Citrin D. E. Somerville R. P. Wunderlich J. R. Danforth D. N. (2016). Randomized, Prospective Evaluation Comparing Intensity of Lymphodepletion before Adoptive Transfer of Tumor-Infiltrating Lymphocytes for Patients with Metastatic Melanoma. J. Clin. Oncol. 34, 2389–2397. 10.1200/JCO.2016.66.7220 27217459 PMC4981979

[B13] Grasso C. S. Giannakis M. Wells D. K. Hamada T. Mu X. J. Quist M. (2018). Genetic Mechanisms of Immune Evasion in Colorectal Cancer. Cancer Discov. 8, 730–749. 10.1158/2159-8290.CD-17-1327 29510987 PMC5984687

[B14] Gross G. Waks T. Eshhar Z. (1989). Expression of Immunoglobulin-T-Cell Receptor Chimeric Molecules as Functional Receptors with Antibody-type Specificity. Proc. Natl. Acad. Sci. U S A. 86, 10024–10028. 10.1073/pnas.86.24.10024 2513569 PMC298636

[B15] Herlyn D. Herlyn M. Steplewski Z. Koprowski H. (1979). Monoclonal Antibodies in Cell-Mediated Cytotoxicity against Human Melanoma and Colorectal Carcinoma. Eur. J. Immunol. 9, 657–659. 10.1002/eji.1830090817 499332

[B16] Hess Michelini R. Doedens A. L. Goldrath A. W. Hedrick S. M. (2013). Differentiation of CD8 Memory T Cells Depends on Foxo1. J. Exp. Med. 210, 1189–1200. 10.1084/jem.20130392 23712431 PMC3674697

[B17] Ishiguro K. Yan I. K. Lewis-Tuffin L. Patel T. (2020). Targeting Liver Cancer Stem Cells Using Engineered Biological Nanoparticles for the Treatment of Hepatocellular Cancer. Hepatol. Commun. 4, 298–313. 10.1002/hep4.1462 32025612 PMC6996342

[B18] Joshi N. S. Cui W. Chandele A. Lee H. K. Urso D. R. Hagman J. (2007). Inflammation Directs Memory Precursor and Short-Lived Effector CD8(+) T Cell Fates via the Graded Expression of T-Bet Transcription Factor. Immunity 27, 281–295. 10.1016/j.immuni.2007.07.010 17723218 PMC2034442

[B19] Kallies A. Xin A. Belz G. T. Nutt S. L. (2009). Blimp-1 Transcription Factor Is Required for the Differentiation of Effector CD8(+) T Cells and Memory Responses. Immunity 31, 283–295. 10.1016/j.immuni.2009.06.021 19664942

[B20] Liang K. H. Tso H. C. Hung S. H. Kuan II Lai J. K. Ke F. Y. (2018). Extracellular Domain of EpCAM Enhances Tumor Progression through EGFR Signaling in colon Cancer Cells. Cancer Lett. 433, 165–175. 10.1016/j.canlet.2018.06.040 29981429

[B21] Luke J. J. Bao R. Sweis R. F. Spranger S. Gajewski T. F. (2019). WNT/β-catenin Pathway Activation Correlates with Immune Exclusion across Human Cancers. Clin. Cancer Res. 25, 3074–3083. 10.1158/1078-0432.CCR-18-1942 30635339 PMC6522301

[B22] Mariathasan S. Turley S. J. Nickles D. Castiglioni A. Yuen K. Wang Y. (2018). TGFβ Attenuates Tumour Response to PD-L1 Blockade by Contributing to Exclusion of T Cells. Nature 554, 544–548. 10.1038/nature25501 29443960 PMC6028240

[B23] Masson F. Minnich M. Olshansky M. Bilic I. Mount A. M. Kallies A. (2013). Id2-mediated Inhibition of E2A Represses Memory CD8+ T Cell Differentiation. J. Immunol. 190, 4585–4594. 10.4049/jimmunol.1300099 23536629 PMC3631715

[B24] McLane L. M. Banerjee P. P. Cosma G. L. Makedonas G. Wherry E. J. Orange J. S. (2013). Differential Localization of T-Bet and Eomes in CD8 T Cell Memory Populations. J. Immunol. 190, 3207–3215. 10.4049/jimmunol.1201556 23455505 PMC3608800

[B25] McQuade R. M. Stojanovska V. Bornstein J. C. Nurgali K. (2017). Colorectal Cancer Chemotherapy: The Evolution of Treatment and New Approaches. Curr. Med. Chem. 24, 1537–1557. 10.2174/0929867324666170111152436 28079003

[B26] Momburg F. Moldenhauer G. Hämmerling G. J. Möller P. (1987). Immunohistochemical Study of the Expression of a Mr 34,000 Human Epithelium-specific Surface Glycoprotein in normal and Malignant Tissues. Cancer Res. 47, 2883–2891. 3552208

[B27] Neelapu S. S. Tummala S. Kebriaei P. Wierda W. Gutierrez C. Locke F. L. (2018). Chimeric Antigen Receptor T-Cell Therapy - Assessment and Management of Toxicities. Nat. Rev. Clin. Oncol. 15, 47–62. 10.1038/nrclinonc.2017.148 28925994 PMC6733403

[B28] Parente-Pereira A. C. Burnet J. Ellison D. Foster J. Davies D. M. van der Stegen S. (2011). Trafficking of CAR-Engineered Human T Cells Following Regional or Systemic Adoptive Transfer in SCID Beige Mice. J. Clin. Immunol. 31, 710–718. 10.1007/s10875-011-9532-8 21505816

[B29] Patriarca C. Macchi R. M. Marschner A. K. Mellstedt H. (2012). Epithelial Cell Adhesion Molecule Expression (CD326) in Cancer: a Short Review. Cancer Treat. Rev. 38, 68–75. 10.1016/j.ctrv.2011.04.002 21576002

[B30] Pickup M. Novitskiy S. Moses H. L. (2013). The Roles of TGFβ in the Tumour Microenvironment. Nat. Rev. Cancer 13, 788–799. 10.1038/nrc3603 24132110 PMC4025940

[B31] Prlic M. Bevan M. J. (2011). Cutting Edge: β-catenin Is Dispensable for T Cell Effector Differentiation, Memory Formation, and Recall Responses. J. Immunol. 187, 1542–1546. 10.4049/jimmunol.1100907 21724993 PMC3150307

[B32] Rahir G. Moser M. (2012). Tumor Microenvironment and Lymphocyte Infiltration. Cancer Immunol. Immunother. 61, 751–759. 10.1007/s00262-012-1253-1 22488275 PMC11028584

[B33] Ramos C. A. Dotti G. (2011). Chimeric Antigen Receptor (CAR)-engineered Lymphocytes for Cancer Therapy. Expert Opin. Biol. Ther. 11, 855–873. 10.1517/14712598.2011.573476 21463133 PMC3107373

[B34] Roessler S. Budhu A. Wang X. W. (2014). Deciphering Cancer Heterogeneity: the Biological Space. Front Cel Dev Biol 2, 12. 10.3389/fcell.2014.00012 PMC4207029 25364720

[B35] Romero J. M. Grünwald B. Jang G. H. Bavi P. P. Jhaveri A. Masoomian M. (2020). A Four-Chemokine Signature Is Associated with a T-Cell-Inflamed Phenotype in Primary and Metastatic Pancreatic Cancer. Clin. Cancer Res. 26, 1997–2010. 10.1158/1078-0432.CCR-19-2803 31964786

[B36] Ruella M. Barrett D. M. Kenderian S. S. Shestova O. Hofmann T. J. Perazzelli J. (2016). Dual CD19 and CD123 Targeting Prevents Antigen-Loss Relapses after CD19-Directed Immunotherapies. J. Clin. Invest. 126, 3814–3826. 10.1172/JCI87366 27571406 PMC5096828

[B37] Schietinger A. Philip M. Krisnawan V. E. Chiu E. Y. Delrow J. J. Basom R. S. (2016). Tumor-Specific T Cell Dysfunction Is a Dynamic Antigen-Driven Differentiation Program Initiated Early during Tumorigenesis. Immunity 45, 389–401. 10.1016/j.immuni.2016.07.011 27521269 PMC5119632

[B38] Schmidt M. Hasenclever D. Schaeffer M. Boehm D. Cotarelo C. Steiner E. (2008). Prognostic Effect of Epithelial Cell Adhesion Molecule Overexpression in Untreated Node-Negative Breast Cancer. Clin. Cancer Res. 14, 5849–5855. 10.1158/1078-0432.CCR-08-0669 18794096

[B39] Sotillo E. Barrett D. M. Black K. L. Bagashev A. Oldridge D. Wu G. (2015). Convergence of Acquired Mutations and Alternative Splicing of CD19 Enables Resistance to CART-19 Immunotherapy. Cancer Discov. 5, 1282–1295. 10.1158/2159-8290.CD-15-1020 26516065 PMC4670800

[B40] Spranger S. Bao R. Gajewski T. F. (2015). Melanoma-intrinsic β-catenin Signalling Prevents Anti-tumour Immunity. Nature 523, 231–235. 10.1038/nature14404 25970248

[B41] Trzpis M. McLaughlin P. M. de Leij L. M. Harmsen M. C. (2007). Epithelial Cell Adhesion Molecule: More Than a Carcinoma Marker and Adhesion Molecule. Am. J. Pathol. 171, 386–395. 10.2353/ajpath.2007.070152 17600130 PMC1934518

[B42] Weber B. N. Chi A. W. Chavez A. Yashiro-Ohtani Y. Yang Q. Shestova O. (2011). A Critical Role for TCF-1 in T-Lineage Specification and Differentiation. Nature 476, 63–68. 10.1038/nature10279 21814277 PMC3156435

[B43] Yamashita T. Budhu A. Forgues M. Wang X. W. (2007). Activation of Hepatic Stem Cell Marker EpCAM by Wnt-Beta-Catenin Signaling in Hepatocellular Carcinoma. Cancer Res. 67, 10831–10839. 10.1158/0008-5472.CAN-07-0908 18006828

[B44] Zhang D. Yang L. Liu X. Gao J. Liu T. Yan Q. (2020). Hypoxia Modulates Stem Cell Properties and Induces EMT through N-Glycosylation of EpCAM in Breast Cancer Cells. J. Cel Physiol 235, 3626–3633. 10.1002/jcp.29252 PMC13482652 31584203

[B45] Zhong X. S. Matsushita M. Plotkin J. Riviere I. Sadelain M. (2010). Chimeric Antigen Receptors Combining 4-1BB and CD28 Signaling Domains Augment PI3kinase/AKT/Bcl-XL Activation and CD8+ T Cell-Mediated Tumor Eradication. Mol. Ther. 18, 413–420. 10.1038/mt.2009.210 19773745 PMC2839303

