# Supplementary material for: Targeting Wnt Signaling in the Tumor Immune Microenvironment to Enhancing EpCAM CAR T-Cell therapy
Source: Front Pharmacol. 2021 Nov 1;12:724306. doi: 10.3389/fphar.2021.724306 (PMC8591126; doi:10.3389/fphar.2021.724306)
Supplement: Supplementary file 1 [file DataSheet1.PDF]

A

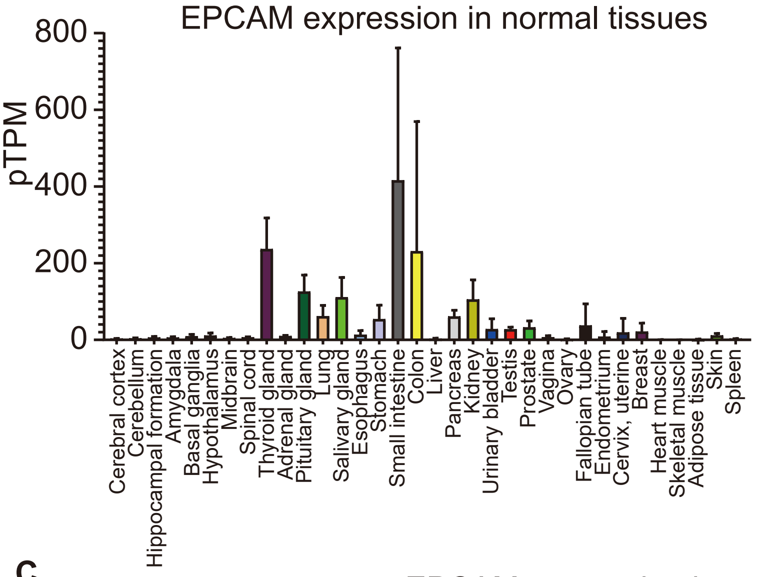

B

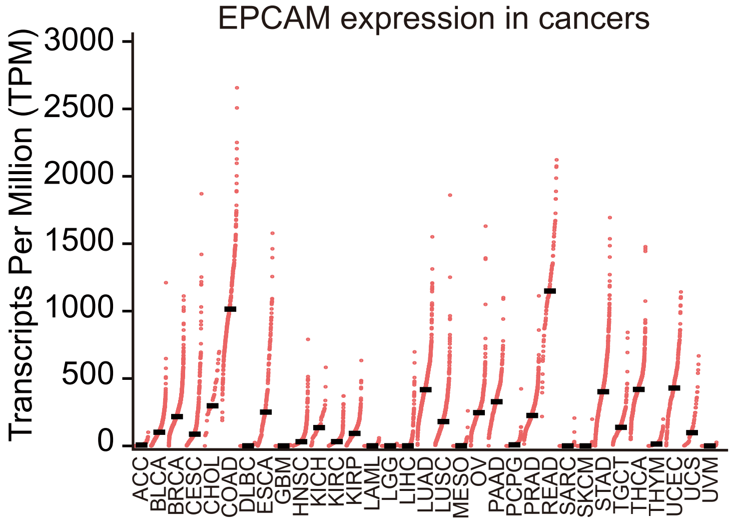

C

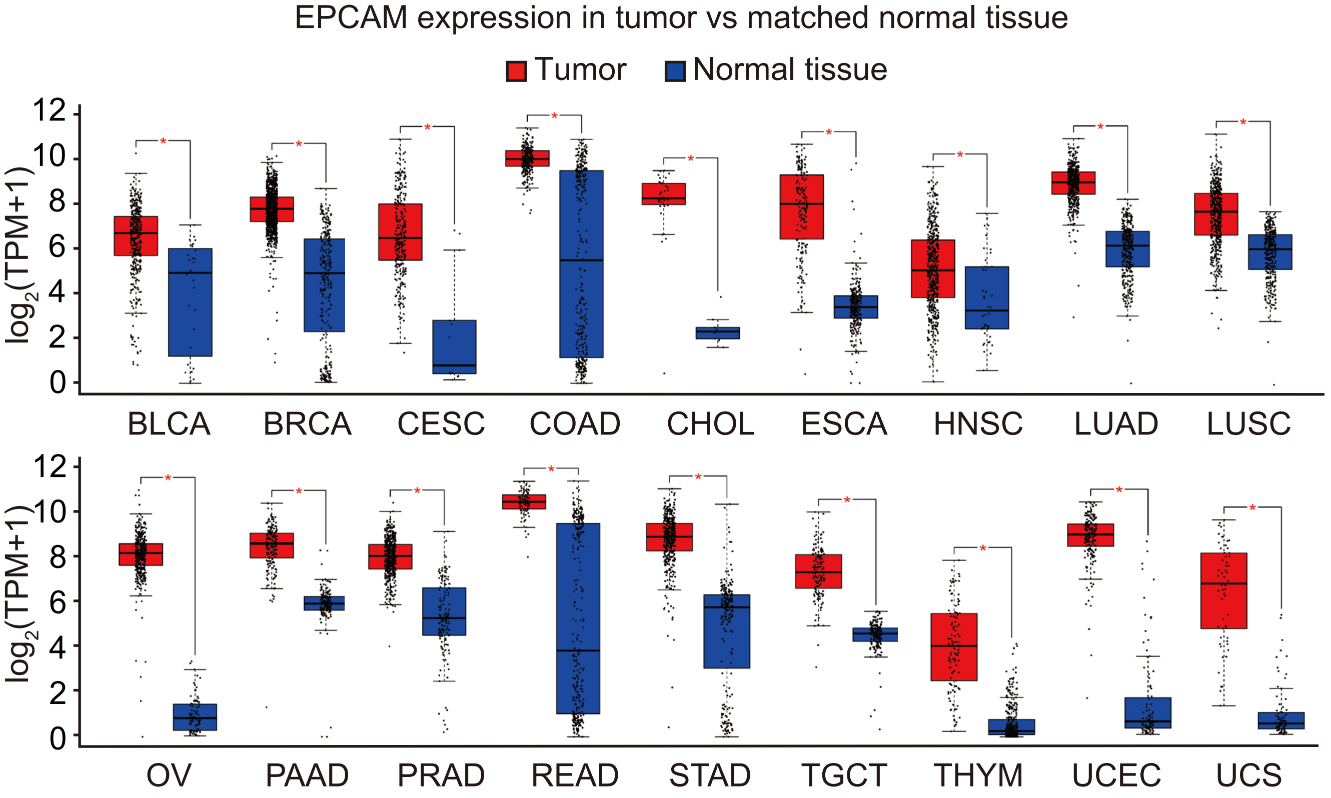

D

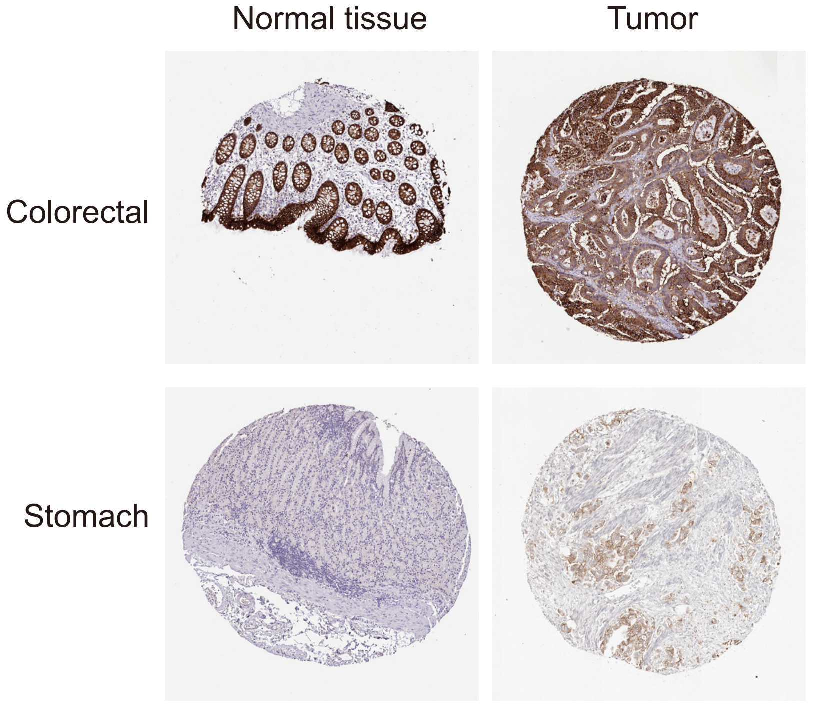

Figure S1

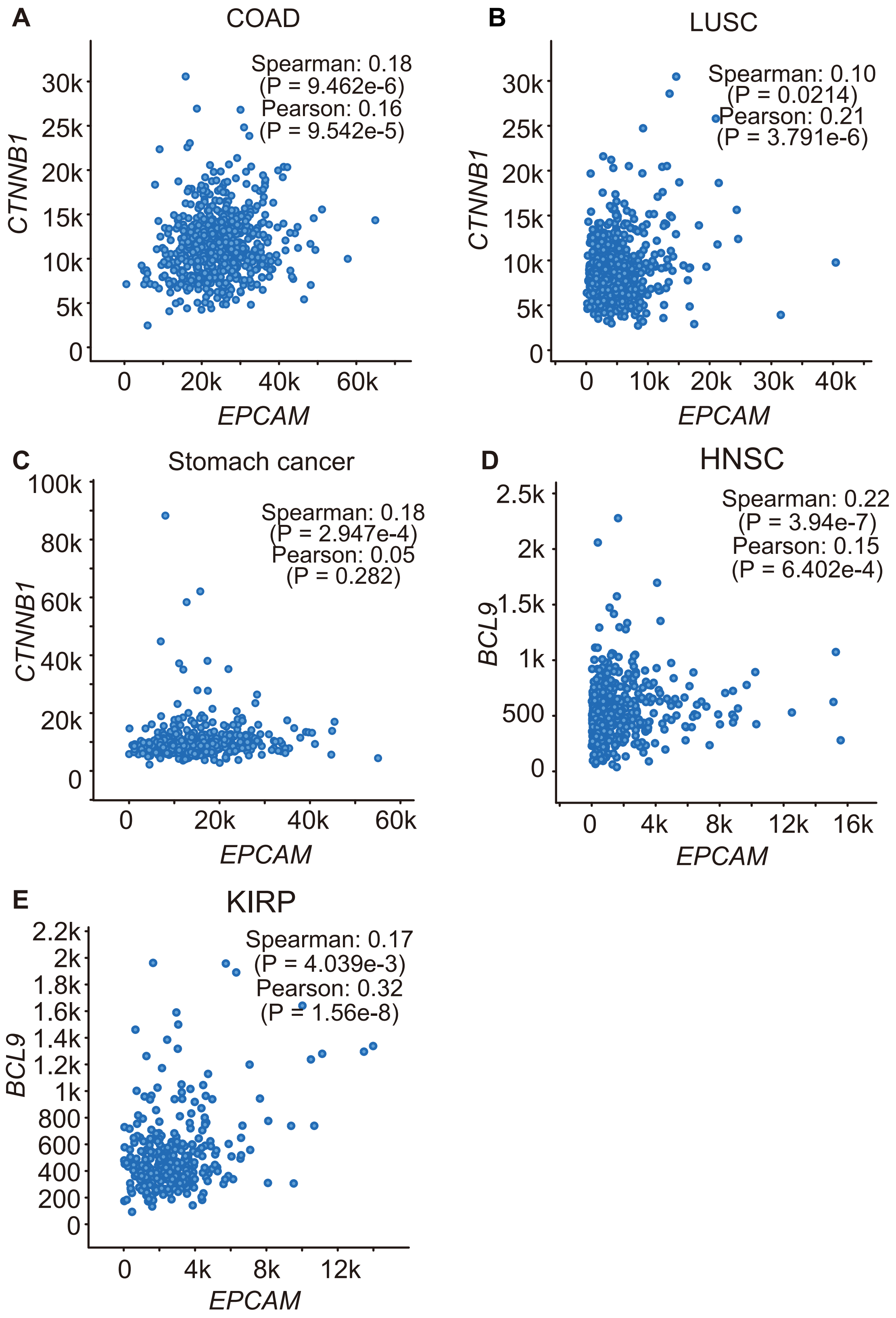
A
COAD
Spearman: 0.18
(P = 9.462e-6)
Pearson: 0.16
(P = 9.542e-5)
CTNNB1
EPCAM
B
LUSC
Spearman: 0.10
(P = 0.0214)
Pearson: 0.21
(P = 3.791e-6)
CTNNB1
EPCAM
C
Stomach cancer
Spearman: 0.18
(P = 2.947e-4)
Pearson: 0.05
(P = 0.282)
CTNNB1
EPCAM
D
HNSC
Spearman: 0.22
(P = 3.94e-7)
Pearson: 0.15
(P = 6.402e-4)
BCL9
EPCAM
E
KIRP
Spearman: 0.17
(P = 4.039e-3)
Pearson: 0.32
(P = 1.56e-8)
BCL9
EPCAM

Figure S2

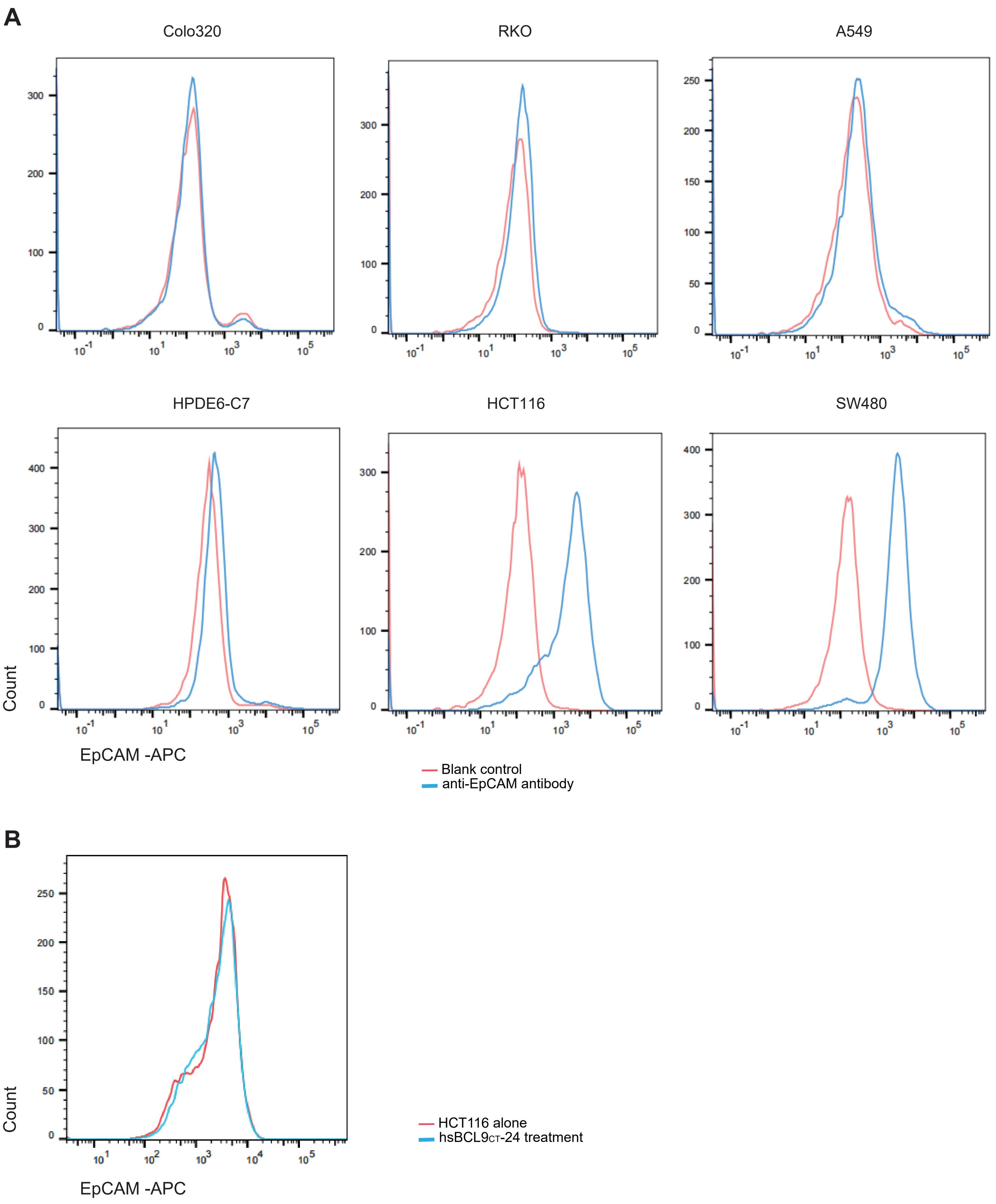
A
Colo320
RKO
A549
HPDE6-C7
HCT116
SW480
Count
EpCAM -APC
Blank control
anti-EpCAM antibody
B
Count
EpCAM -APC
HCT116 alone
hsBCL9$_{CT}$-24 treatment

Figure S3
